# Human Glioblastoma Multiforme: p53 Reactivation by a Novel MDM2 Inhibitor

**DOI:** 10.1371/journal.pone.0072281

**Published:** 2013-08-19

**Authors:** Barbara Costa, Sara Bendinelli, Pamela Gabelloni, Eleonora Da Pozzo, Simona Daniele, Fabrizio Scatena, Renato Vanacore, Pietro Campiglia, Alessia Bertamino, Isabel Gomez-Monterrey, Daniela Sorriento, Carmine Del Giudice, Guido Iaccarino, Ettore Novellino, Claudia Martini

**Affiliations:** 1 Department of Pharmacy, University of Pisa, Pisa, Italy; 2 U.O. Immunohaematology 2, Cisanello Hospital, Pisa, Italy; 3 Department of Pharmaceutical Science, Division of BioMedicine, University of Salerno, Salerno, Italy; 4 Department of Pharmaceutical and Toxicological Chemistry, University of Naples Federico II, Naples, Italy; 5 Department of Advanced Biomedical Sciences, University of Naples Federico II, Naples, Italy; 6 Department of Medicine and Surgery, University of Salerno, Salerno, Italy; 7 IRCCS Multimedica, Milano, Italy; Complutense University, Spain

## Abstract

Cancer development and chemo-resistance are often due to impaired functioning of the p53 tumor suppressor through genetic mutation or sequestration by other proteins. In glioblastoma multiforme (GBM), p53 availability is frequently reduced because it binds to the Murine Double Minute-2 (MDM2) oncoprotein, which accumulates at high concentrations in tumor cells. The use of MDM2 inhibitors that interfere with the binding of p53 and MDM2 has become a valid approach to inhibit cell growth in a number of cancers; however little is known about the efficacy of these inhibitors in GBM. We report that a new small-molecule inhibitor of MDM2 with a spirooxoindolepyrrolidine core structure, named ISA27, effectively reactivated p53 function and inhibited human GBM cell growth *in vitro* by inducing cell cycle arrest and apoptosis. In immunoincompetent BALB/c nude mice bearing a human GBM xenograft, the administration of ISA27 *in vivo* activated p53, inhibited cell proliferation and induced apoptosis in tumor tissue. Significantly, ISA27 was non-toxic in an *in vitro* normal human cell model and an *in vivo* mouse model. ISA27 administration in combination with temozolomide (TMZ) produced a synergistic inhibitory effect on GBM cell viability *in vitro*, suggesting the possibility of lowering the dose of TMZ used in the treatment of GBM. In conclusion, our data show that ISA27 releases the powerful antitumor capacities of p53 in GBM cells. The use of this MDM2 inhibitor could become a novel therapy for the treatment of GBM patients.

## Introduction

Glioblastoma multiforme (GBM) is the most common and aggressive brain tumor in humans, and despite technical advances in neurosurgery and clinical neuro-oncology, the prognosis for GBM patients remains very poor [1]–[8]. Most patients die within one year of diagnosis and are generally insensitive to current therapeutic genotoxic interventions [1]–[8]. In the majority of GBM cases, resistance to such genotoxic modalities has been attributed to the attenuation of p53 function by alterations within the p53 signalling axis, including the overexpression of Murine Double Minute-2 (MDM2) [9]–[13]. The MDM2 oncoprotein, a major physiological negative regulator of p53, can bind to the p53 transactivation domain and interfere with the transcriptional regulatory mechanisms of p53 [9]–[13]. MDM2 is also an E3 ubiquitin ligase that promotes p53 proteasomal degradation. For this reason, inhibition of the interaction between MDM2 and p53 to reactivate endogenous p53 activity offers the opportunity for therapeutic intervention, particularly in GBMs. In GBMs, the p53 gene is relatively infrequently mutated; however, wild-type p53 remains dysfunctional due to overexpressed MDM2 [9], [10].

Intensive work on different classes of MDM2 inhibitors has proven their therapeutic utility as activators of p53 in multiple tumor models [14]–[17]. Indeed, it has been demonstrated that a number of small-molecule MDM2 inhibitors can disrupt the MDM2-p53 interaction, release p53 from negative control and activate the p53 pathway, leading to cell cycle arrest and apoptosis in a number of solid cancers and haematological malignancies [14]–[17]. Moreover, many laboratories have shown that MDM2 inhibitors can synergise with conventional chemotherapeutic agents, resulting in enhanced efficacy [18]–[22]. Interestingly, MDM2 inhibitors have been reported to induce cancer cell apoptosis even without the concomitant application of genotoxic stimuli [17], [18], [20]. Little is known about the effects of MDM2 inhibitors on the *in vitro* growth of GBM cells. Recently, Nutlin-3, the first potent MDM2 small-molecule inhibitor identified [23], and new D-peptide derivatives [14], [24] were reported to be effective at inhibiting GBM cell growth *in vitro* [14], [24], [25], suggesting the validity of this experimental approach for the treatment of GBM.

In the present study, we investigated the responsiveness of human GBM cell lines to a novel small-molecule MDM2 inhibitor with a spirooxoindolepyrrolidine core structure, named ISA27, which has been recently shown by nuclear magnetic resonance (NMR) analysis to efficiently dissociate the reconstituted human MDM2-p53 complex [26]. Consistently, ISA27 activated the p53 pathway in GBM cells and elicited the dose- and time-dependent inhibition of cell growth. ISA27 induced apoptosis and evoked cellular senescence, indicating that ISA27 promotes a pleiotropic anticancer effect in the GBM cells. The administration of ISA27 *in vivo* efficiently inhibited tumor growth in nude mice bearing a human GBM xenograft. Significantly, ISA27 was non-toxic both *in vitro* in a normal human cell model and *in vivo* in a mouse model.

## Materials and Methods

### 1. Materials

ISA27 was synthesised as previously reported [26]. Nutlin-3, carbonylcyanide-m-chlorophenylhydrazone (CCCP), Nonidet P-40 (NP-40) and cycloheximide (CHX) were obtained from Sigma–Aldrich, Milano, Italy. Propidium iodide (PI) and the fluorescent dye, 5,50,6,60-tetrachloro-1,10,3,30-tetraethylbenzimidazolcarbocyanine iodide (JC-1) were obtained from Molecular Probes, Invitrogen, Milano, Italy. The 3-(4,5-dimethylthiazol-2-yl)-5-(3-carboxymethoxyphenyl)-2-(4-sulfophenyl)-2H-tetrazolium (MTS) assay kit was from Promega Italia, Milano, Italy. The RNeasy® Mini Kit was from Qiagen, Milano, Italy and the ProtoScript® cDNA Synthesis Kit was obtained from Biolabs, Euroclone, Milano, Italy. The mitochondrial fractionation Active Motif® Kit was purchased from Active Motif, Rixensart, Belgium and the Platinum Human Cytochrome C ELISA was obtained from Bender MedSystems GmbH, Vienna, Austria. Antibodies against p53 (FL-393) and MDM2 (C-18) were from Santa Cruz Biotechnology.

### 2. GBM Cell Line Culture and Preparation of Cells from Peripheral Blood

The U87MG, T98G and U343MG cell lines were obtained from the National Institute for Cancer Research of Genoa (Italy), American Type Culture Collection (USA) and Cell Lines Service GmbH (Germany), respectively. Each cell line was monitored for DNA profiling. The U87MG and T98G cells were cultured in RPMI medium and Minimum essential medium Eagle, respectively, supplemented with 10% FBS, 2 mM L-glutamine, 100 U/mL penicillin, 100 mg/mL streptomycin and 1% non-essential amino acids at 37°C in 5% CO_2_. The U343MG cells were cultured in Minimum essential medium Eagle with 2 mM L-glutamine and Earle's BSS adjusted to contain 1.5 g/L sodium bicarbonate and supplemented with 10% FBS, 100 U/mL penicillin, 100 mg/mL streptomycin, 1% non-essential amino acids and 1.0 mM sodium pyruvate at 37°C in 5% CO_2_.

Mononuclear cell preparation was performed according to the method of Boyum [27]. The final cell pellet was suspended in complete RPMI 1690 media supplemented with 15% FBS, 2 mM L-glutamine, 100 U/mL penicillin and 100 mg/mL streptomycin. To evaluate cell populations, random cell samples (n = 7) were employed for flow cytometric analysis.

### 3. Cell Treatments

The human GBM cells were seeded at 5,000 cells/cm^2^. After 24 h, the culture medium was replaced with fresh medium containing MDM2 inhibitor solubilised in DMSO for the indicated incubation times. DMSO was added to control cells (<1% v/v). For short-term treatment (up to 24 h), GBM cells were incubated with increasing concentrations or a fixed concentration of MDM2 inhibitor corresponding to the concentration that inhibited 50% (IC_50_ value) of GBM cell survival/growth; for long-term treatment (up to 5 days), U87MG cells and lymphomonocytes were incubated with 2.5 µM ISA27 or 10 µM Nutlin-3.

### 4. Analysis of p53 Protein Stabilisation

Following GBM cell treatment with MDM2 inhibitor, stabilisation of the p53 protein was evaluated as previously described [28]–[30]. Briefly, GBM cells were treated with DMSO (control) or MDM2 inhibitor for 4, 6, 8 and 12 h and then lysed for 60 min at 4°C by adding RIPA buffer (9.1 mM NaH_2_PO_4_, 1.7 mM Na_2_HPO_4_, 150 mM NaCl, pH 7.4, 0.5% sodium deoxycholate, 1% Nonidet P-40, 0.1% SDS and a protease inhibitor cocktail). Equal amounts of cell extracts (40 µg) from MDM2 inhibitor-treated and untreated cells were diluted in Laemmli solution, resolved by SDS-PAGE (8.5%), transferred to PVDF membranes and probed overnight at 4°C with a primary anti-p53 (FL-393, 1∶500) antibody. The primary antibody was detected using anti-rabbit IgG light chains conjugated to peroxidase (diluted 1∶10,000). The peroxidase was detected using a chemiluminescent substrate (ECL, Perkin Elmer). Western blot analysis was also performed using lysates from MDM2 inhibitor-treated and untreated GBM cells in the absence and presence of the protein synthesis inhibitor CHX (50 µM).

The relative quantification of p53 mRNA was performed by real-time reverse transcription polymerase chain reaction (real-time RT-PCR) as previously described [31] in MDM2 inhibitor-treated and untreated GBM cells. In brief, total RNA was isolated using the RNeasy® Mini Kit. The purity of the RNA samples was determined by measuring the absorbance at 260∶280 nm. cDNA synthesis was performed with 500 ng of RNA using the Quantitect® reverse transcriptase kit. The primers used for the RT-PCR were designed to span intron/exon boundaries to ensure that products did not include genomic DNA (see Table 1). The RT-PCR reactions consisted of 12.5 µL of Brilliant® II SYBR® Green premix, 2.5 µL of both the forward and reverse primers (at a 10 µM concentration), 3 µL of cDNA and 4.5 µL of H_2_O. All reactions were performed for 40 cycles using the following temperature profiles: 98°C for 30 seconds (initial denaturation); 55°C for 30 seconds (annealing) and 72°C for 3 seconds (extension). β-actin was used as the housekeeping gene. PCR specificity was determined using both a melting curve analysis and gel electrophoresis, and the data were analysed by the standard curve method. p53 mRNA levels for each sample were normalised against β-actin mRNA levels, and relative expression was calculated using the Ct value.

**Table 1:** Real-time RT-PCR: primer nucleotide sequences, position in mRNA sequence and amplicon length.

Name and other designations	Access. numb.mRNA length	Primer nucleotide sequences	Primer position inmRNA sequence	Tm(°C)	Ampliconlenght
Mdm2, p53 E3 ubiquitin protein ligasehomolog (mouse) (MDM2)	NM_002392.47472 pb	FOR: 5′-TCTAGGAGATTTGTTTGGCGT-3′;REV: 5′-TCACAGATGTACCTGAGTCC-3	Exon 4 (548–568)Exon 6 (653–672)	6060	125 pb
Cyclin-dependent kinase inhibitor 1A (p21,Cip1) (CDKN1A)	NM_0003892175 pb	FOR: 5′-TGCCGAAGTCAGTTCCTTG -3′;REV: 5′-CATGGGTTCTGACGGACATC-3′	Exon 3 (50–68)Exon 4 (144–163)	6062	134 pb
Bcl2 binding component 3 (BBC3), JFY1, PUMA	NM_0011272401839 pb	FOR: 5′-GAGGAGGAACAGTGGGC-3′;REV: 5′- CTAATTGGGCTCCATCTCGG-3′	Exon 4 (652–668)Exon 5 (830–849)	5662	198 pb
Tumor protein p53, antigen NYCO-13, cellulartumor antigen p53, p53 tumor suppressor	NM_0011261122588 pb	FOR: 5′CTTTGAGGTGCGTGTTTGTG-3′;REV: 5′ GTGGTTTCTTCTTTGGCTGG-3′	Exon 8 (1006–1025)Exon 9 (1147–1166)	6060	161 pb
Actin,beta (ACTB)	NM_001101.31852 pb	FOR: 5′-GCACTCTTCCAGCCTTCCTTCC-3′REV-5′-GAGCCGCCGATCCACACG-3′	Exon 4 (862–884)Exon 6 (1097–1115)	7062	254 pb

The amount of p53-MDM2 complex was determined using co-immunoprecipitation experiments; U87MG cells were treated with DMSO (control) or 2.5 µM ISA27 or 10 µM Nutlin-3 for 8 h. One milligram of cell lysates, obtained as described above, was precleared with protein A-Sepharose (1 h at 4°C) to precipitate and eliminate IgG. Samples were then centrifuged for 10 min at 4°C (14,000×g). The supernatants were incubated with an anti-MDM2 antibody (5 µg/sample) overnight at 4°C under constant rotation and then immunoprecipitated with protein A-Sepharose (2 h at 4°C). After washing, the immunocomplexes were resuspended in Laemmli solution and boiled for 5 min, resolved by SDS-PAGE (8.5%), transferred to PVDF membranes and probed overnight at 4°C with primary antibodies to p53 (FL-393, 1∶500) or MDM2 (C-18, 1∶500) as described above.

### 5. Relative mRNA Quantification of p53 Target Genes

The relative mRNA quantification of p53 target genes was performed by real-time RT-PCR as described above. Specific primers for amplification of p21, MDM2 and PUMA mRNA were designed to include intron/exon boundaries and are reported in Table 1.

### 6. Cell Survival/growth Analysis

The effects of MDM2 inhibitor treatment on U87MG cell survival/growth were determined by MTS conversion and Trypan blue exclusion assays [32], [33]. The MTS assay was used to establish the MDM2 inhibitor concentration that inhibited 50% (IC_50_ value) of GBM cell survival/growth after 24 h of treatment and was performed according to the manufacturer’s instructions. Sigmoidal dose-response curves were generated using GraphPad Prism 4 software (GraphPad Software Inc., San Diego, CA), from which the IC_50_ values were derived. Trypan blue dye exclusion was used to examine the long-term effects of MDM2 inhibitor treatment on U87MG cell and lymphomonocyte viability at the indicated times.

### 7. Cell Cycle Analysis

Cell cycle analysis was performed by flow cytometry (FACSCalibur flow cytometer, Becton Dickinson, USA) [32], [34]. Briefly, after treatment, detached and trypsinized adherent cells were collected by centrifugation at 200×*g* for 5 min. Pellets were gently suspended in 500 µl of a hypotonic fluorochrome solution [PI 50 µg/ml in 0.1% (w/v) sodium citrate plus 0.01% (v/v) NP-40 and 10 µg/ml DNase-free RNase in bi-distilled water]. After incubation in the dark at 37°C in 5% CO_2_ for 30 min, cell samples were stored on ice and analysed for DNA content on a logarithmic scale by flow cytometry. Ten thousand events per sample were acquired. The percentage of PI-stained untreated and ΜDM2 inhibitor-treated cells was measured in each cell cycle phase using the ModFit LT software program.

### 8. Cellular Senescence Assays

The senescence marker, Senescence-associated beta-galactosidase (SA-β-Gal), was detected as previously described [35], and the cells were then washed in PBS (1×) and photographed at 100× magnification. Images from random light microscopic fields (5 fields per well) were captured, and the cells were counted using ImageJ software (ImageJ Software, version 1.41o; USA). The average cell size was assessed using the Scepter 2.0 handheld automated cell counter (Millipore, Billerica, MA, USA).

### 9. Analysis of Apoptotic Parameters

#### Mitochondrial membrane potential (Δψm)

Changes in Δ*ψ*m were assessed using the fluorescent dye, JC-1, as previously described [32], [34], and fluorescence was analysed by fluocytometry. As a positive control, a cell pellet was incubated with the uncoupling agent, CCCP (50 µM).

#### Cytosolic cytochrome C release from mitochondria

The cytosolic fraction from untreated and MDM2 inhibitor-treated cells was obtained using the Active Motif® mitochondrial fractionation kit according to the manufacturer’s instructions. The cytosolic protein content was determined as previously described [36], and the cytochrome C content was determined using the Platinum Human Cytochrome C ELISA. The minimum detectable dose of cytochrome C was 0.05 ng/mL.

#### DNA fragmentation analysis

DNA fragmentation was estimated by evaluating PI staining of DNA using FACS. The percentage of apoptotic cells was estimated as previously described [32], [34]. The percentage of cells in the sub-G0 fraction was determined using the ModFit LT software program.

### 10. siRNA Mediated Inhibition of p21 Gene Expression

The p21siRNA and control siRNA were obtained from Santa Cruz Biotecnology (Heidelberg, Germany). The p21siRNA was transfected with siRNA transfection reagent to a final concentration of 50 nM, following the manufacturer's protocol. In parallel to each silencing experiment, an ineffective sequence of RNA has been used as negative control. Transfected cells were used 36 h after siRNA transfection. The silence efficacy was verified by both real time PCR and Western blotting analysis. For Western blotting analysis, U87MG cells were lysed and proteins, resolved by SDS-PAGE electrophoresis, were probed with an anti-p21 antibody (H164, sc-756, Santa Cruz Biotecnology). After p21 genetic inhibition, we incubated U87MG cells with ISA27 for 24 h and assessed cell viability, cell cycle profile, Δψm and phosphatidylserine externalization (PE). The measurement of PE was performed by the Muse™ Cell Analyzer using the fluorescent dyes Annexin V and 7-AAD. Annexin-V is used as a probe to detect cells that have expressed phosphatidylserine on the cell surface. 7-AAD is excluded from viable cells, since it does not pass through intact cell membranes. We simultaneously measured the expression of phosphatidylserine on the cell surface and the cellular plasma membrane permeabilization. Briefly, both floating and adherent treated cells were collected, centrifuged at 300×g for 5 minutes and suspended in cell culture medium. Then, a 100 µl aliquot of cell suspension (about 50000 cell/ml) was added to 100 µl of fluorescent reagent and incubated for 10 minutes at room temperature. After the incubation period, the percentages of living, apoptotic and dead cells were acquired and analysed by Muse Cell Analyzer in accordance to the Millipore guidelines.

### 11. In vivo Study Design

Experiments were performed in accordance with NIH guidelines for animal use using 6-week-old BALB/c immunoincompetent nude mice (Charles River). All animals had access to food and water *ad libitum*. For tumor formation, a suspension containing 2×10^6^ U87MG cells in 200 µl of culture medium were injected subcutaneously in the dorsal side of nude mice as previously described [37]. Animals were anaesthetised using 2% isofluorane. We used mice that developed tumors approximately 6 mm in size in 2 weeks. Mice were divided into 3 groups (5 mice/group) and given intra-tumoral or intra-peritoneal injections of the specific treatment (ISA27) twice a week for 2 weeks. In particular, 1 group received intra-tumoral injections of ISA27 at a dosage of 3 mg/kg, and another group received intra-peritoneal injections of 5 mg/kg of the drug; the control group received intra-tumoral or intra-peritoneal injections of saline solution. Tumor growth was measured by caliper twice a week, and volumes were calculated using the formula, V = A×B^2^/2, where A and B are the major and minor axes, respectively, of the tumor. At the end of the treatment period, mice were sacrificed by cervical dislocation, and the tumors were extracted and photographed.

Tumor tissues were examined for cell proliferation and apoptosis by immunohistochemistry and Western blotting analysis. Immunohistochemistry and Paraffin embedded sections were processed for the triple layered immunocytochemical peroxidase anti-peroxidase (PAP) method. Proliferating Cell Nuclear Antigen (PCNA, HPA030522; SigmaAldrich) and cleaved caspase 3 (ab52293; Abcam) antiserum were used as cell proliferation and apoptosis marker, respectively. The peroxidase was revealed in presence of 0.03% hydrogen peroxide and of an electron donor, 2.5% diaminobenzidine, which becomes visible as a brown precipitate. For negative controls, the primary antiserum was omitted. Sections were then viewed with an Eclipse E1000 Fluorescence Microscope (Nikon) and acquired using Sigma Scan Pro software (Jandel). The expression of PCNA and cleaved caspase 3 was measured from digital images using a dedicated software (NIH ImageJ64). For Western blotting analysis, tissues were homogenized in lysis buffer (50 mM Tris-HCl, 150 mM NaCl, 5 M NaCl, 0.25% SDS, 0.25% Sodium Deoxycholate, 1 mM EDTA, pH 7.5), containing 1% of the Proteinase inhibitor Cocktail (Sigma P8340), and centrifuged at 10.000×g for 10 min. Equal amounts (100 µg) of the supernatants were diluted in Laemmli solution, resolved by SDS-PAGE (12.5%), transferred to PVDF membranes and probed overnight at 4°C with primary antibodies speciphic for caspase 3 (sc-7148, Santa Cruz Biotechnology, 1∶100) or phospho-H3 (sc-12927, Santa Cruz Biotechnology, 1∶200).

Tumor tissues were examined to determine p53 activation by Western blot analysis and real-time RT-PCR. For Western blotting analysis, tissues were processed as above described and probed with antibodies against p53 or p21. For real-time RT-PCR analysis, about 20 mg of each tissue’s sample was used for total RNA isolation and retro-transcription as reported for cell samples. Primers for p21, MDM2 and PUMA are reported in Table 1. For the toxicity assay, Masson trichrome staining was performed on paraffin sections from the liver, kidney and lung of mice treated with intra-peritoneal injections of ISA27 and control mice as previously described [38].

### 12. Isobolar Analysis

A graphical assessment of synergy with regard to growth inhibition was performed using isobolographic analysis [39], [40]. In an isobologram, the equi-effective pairs of doses of two drugs (ISA27 and Temozolomide) are represented using rectangular coordinates. In the present study, the dose of ISA27 required to give a 50% effect was plotted on the abscissa, and the iso-effective dose of Temozolomide was plotted on the ordinate. The theoretical additive effect of the two drugs was represented by the straight line connecting the two points. If the experimentally determined data points and their confidence interval fall on this line, the drug effects are additive (no interaction). If the points lie below this line, there is superadditivity (synergy), and if the points lie above this line, there is subadditivity (antagonism).

To determine whether the interaction between the two drugs was synergistic, additive or antagonistic, the theoretical additive IC_50,add_ was estimated from the dose-response curves of each drug administered individually, considering that the observed effect with the combination was the sum of the individual effects of each component. The theoretical IC_50,add_ value was then compared with the experimental IC_50,mix_ to determine whether there was a statistically significant difference [41]. The interaction index, denoted by γ, is an assessment of the degree of synergism or antagonism. The index is defined by the isobolar relationship as follows [42]: γ = a/A+b/B where A and B are the doses of drug A (alone) and B (alone) that give the specified effect, and (a,b) are the combination doses that produce the same effect. The quantities in the equation are obtained from the dose-response curves of drugs A, B, and their combinations. If γ = 1, the interaction is additive; if γ <1, the interaction is super-additive (synergy); and if γ >1, the interaction is sub-additive (antagonism).

### 13. Statistical Analyses

The nonlinear multipurpose curve-fitting program, GraphPad Prism, was used for data analysis and graphic presentations. All data are presented as the means ± SEM. Statistical analysis was performed by one-way analysis of variance (ANOVA) with Bonferroni’s corrected t-test for post-hoc pair-wise comparisons. The densitometric analysis of immunoreactive bands was performed using the ImageJ program. For *in vivo* experimental data, a two-way ANOVA was performed to compare the different parameters among the different groups. P<0.05 was considered statistically significant.

## Results

### 1. Short-term ISA27 Treatment Inhibits GBM Cell Growth/survival

**Figure 1:**
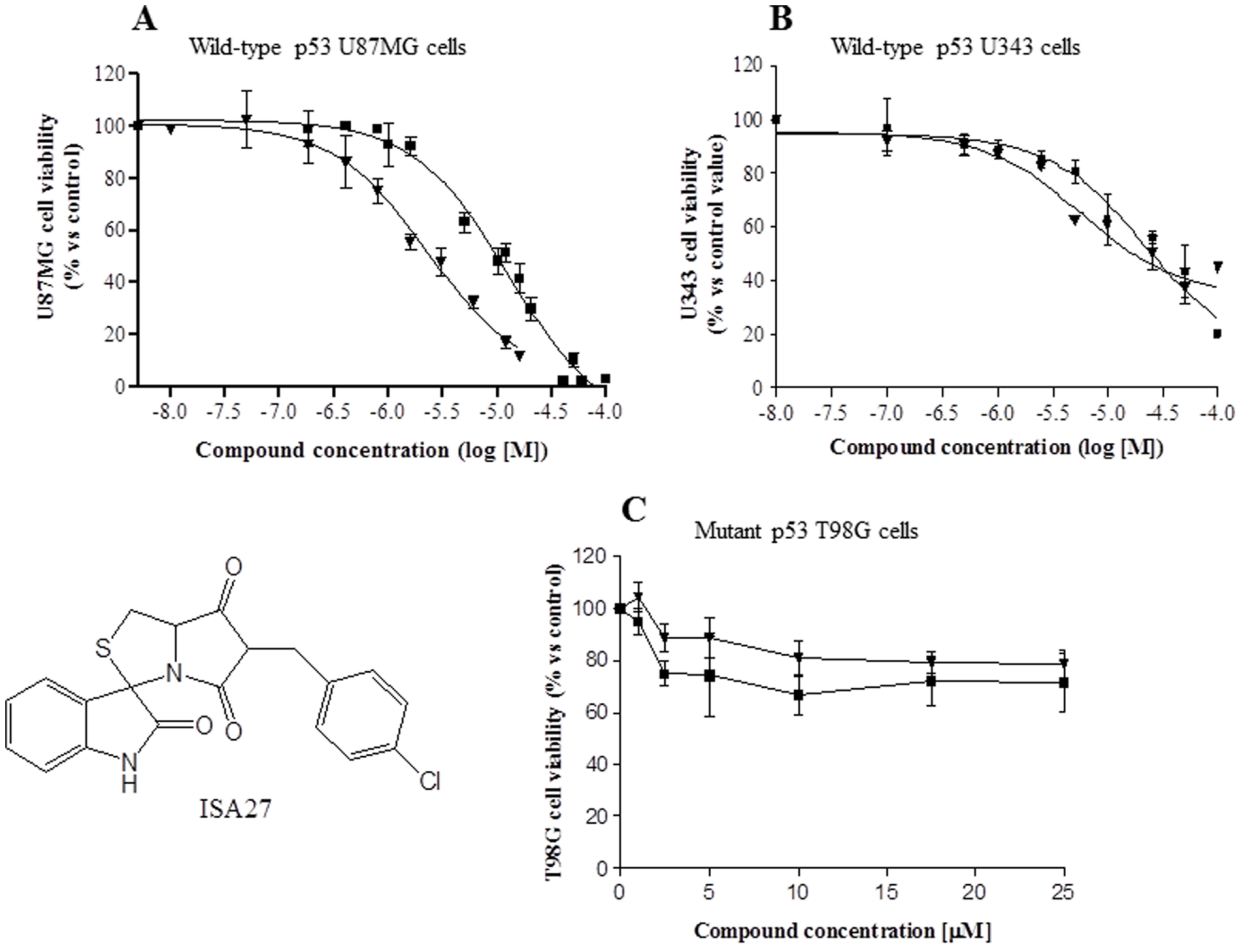
ISA27 inhibits the growth/survival of GBM cell lines expressing wild-type p53. GBM cells with wild-type p53 (U87MG, U343MG) were exposed to a range of ISA27 or Nutlin-3 concentrations for 24 h. After incubation, GBM cell viability was determined by MTS assay. *A and B) Effect of ISA27 on the growth/survival of GBM cell lines expressing wild-type p53:* curves for U87MG and U343MG cell samples were generated using a sigmoidal dose-response curve model (GraphPad Prism 4 software) from which the IC_50_ values were derived. ISA27-treated cells (▾), Nutlin-3-treated cells (▪); *C) Effect of ISA27 on the growth/survival of the GBM T98G cell line expressing mutant p53:* T98G cells were incubated with increasing concentrations of ISA27 or Nutlin-3 (ranging from 1 µM to 25 µM), and cell viability was measured after 24 h by MTS assay. ISA27-treated cells (**▾**), Nutlin-3-treated cells (**▪**). The results are expressed as the percentages of cell viability with respect to the vehicle-treated sample for each cell line; the viability of the vehicle-treated sample was arbitrarily set at 100%. Points, means of three independent experiments performed in duplicate; bars, SEM.

To examine the effects of ISA27 [26] (see Fig. 1 for chemical structure) on GBM cell growth/survival, the concentration at which this molecule inhibited 50% of cell growth/survival (IC_50_ value) after 24 h of treatment was assessed. We used U87MG and U343MG cell lines that overexpress MDM2 and maintain wild-type p53 [43]. ISA27 showed a dose-dependent inhibitory effect on the growth/survival of U87MG and U343MG cells with IC_50_ values of 2.5±0.4 and 5.7±0.9 µM, respectively (the same cell treatment with Nutlin-3 used as a control gave a value of 10.0±2.1 and 22.0±4.6 µM, respectively) (Fig. 1A and B). We also used the GBM cell line T98G, which expresses mutant p53 [43], as a negative control for the effect of ISA27 on GBM cell viability. Treatment of T98G cells with ISA27 did not significantly reduce cell growth/survival, showing approximately 80% of viable cells at the maximum tested concentration (25 µM) (Fig. 1C). The treatment of T98G cells with Nutlin-3 gave a similar result (Fig. 1C).

### 2. Short-term ISA27 Treatment Stabilises p53 Levels and Restores p53 Function in GBM Cells Expressing Wild-type p53

**Figure 2:**
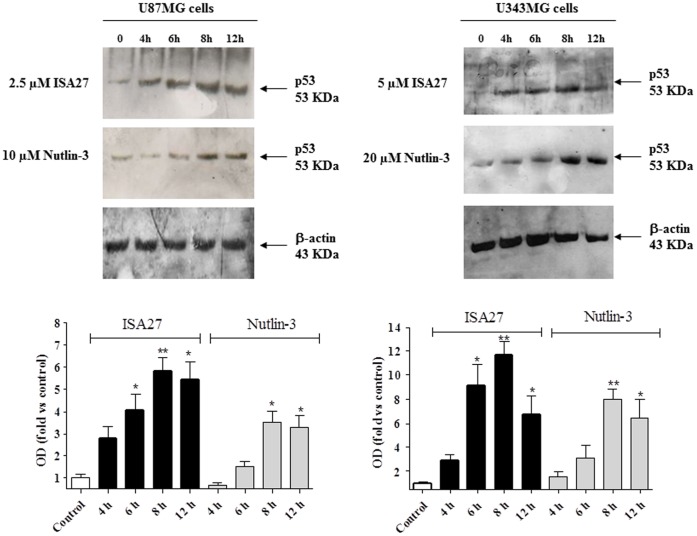
ISA27 increases p53 protein levels in GBM cell lines. GBM cells (U87MG, U343MG) were treated with ISA27 or Nutlin-3 for the indicated incubation times, and protein levels of p53 were analysed in whole-cell lysates by Western blot. One representative Western blot is presented (upper panel) for each cell line. The blots show that the antibody to p53 (FL-393; Santa Cruz Biotechnology) recognised a single specific band at approximately 55 kDa, corresponding to the molecular weight of the p53 protein; β-actin is used as the loading control. Densitometric analyses of Western blots (lower panel) demonstrated that ISA27 induced a significant enhancement of p53 protein levels in U87MG or U343MG cells with a maximal effect at the 8 h incubation time.

We first examined the effect of ISA27 on the levels of p53 in the GBM cells, U87MG and U343MG. Incubation of the GBM cells with ISA27 for 4, 6, 8, and 12 h led to a time-dependent increase in p53 protein levels (Fig. 2). The accumulation of p53 became significant after 6 hours of treatment, reaching its highest level after 8 h (Fig. 2). Nutlin-3 induced a significant accumulation of p53 protein after 8 h and 12 h of treatment (Fig. 2).

**Figure 3:**
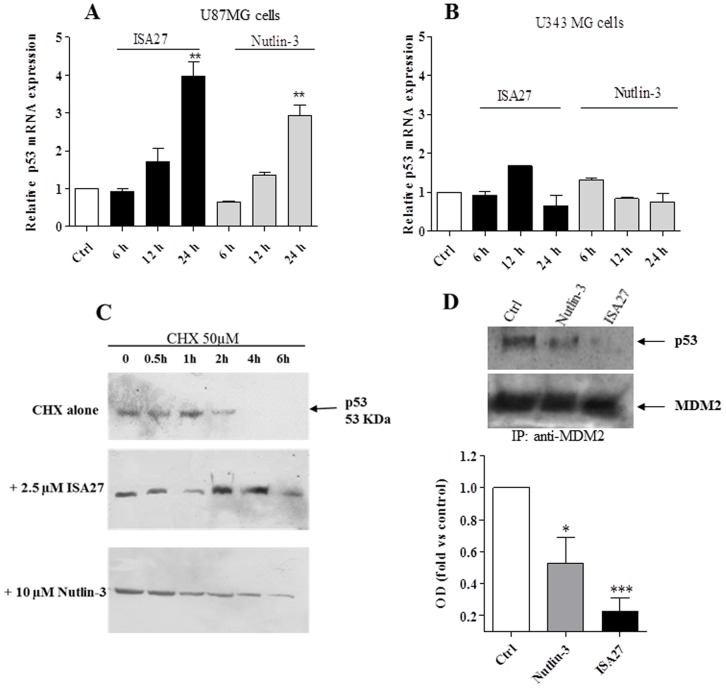
ISA27 induces the stabilisation of p53 protein levels by decreasing the interaction of p53 with MDM2 in GBM cells. *A and B) Effect of ISA27 on p53 gene expression:* U87MG and U343MG cells were treated with ISA27 or Nutlin-3 for 6, 12 and 24 h. After incubation, the relative quantification of p53 mRNA was performed by real-time RT-PCR. In U87MG cells, p53 mRNA levels did not change within 12 h of cell treatment; in U343 MG cells, p53 mRNA levels did not change during any of the incubation times. *3C) ISA27 sustains p53 protein expression in the presence of CHX:* U87MG cells were incubated with 50 µM CHX alone or CHX in combination with ISA27 or Nutlin-3 for 0.5, 1, 2, 4 and 6 h. After incubation, cell lysates were used for Western blot analysis. One representative Western blot is presented for each cell treatment. The inhibition of protein synthesis by treatment with CHX alone blocked p53 protein expression after a 4-h treatment. ISA27 extended the stability of p53 in the presence of CHX. *3D) ISA27 reduces MDM2-p53 complex formation*: U87MG cells were incubated with ISA27 or Nutlin-3 for 8 h followed by immunoprecipitation using an anti-MDM2 antibody. The MDM2-p53 complex and the relative input of the proteins were detected by immunoblot; a significant reduction in MDM2-p53 complex formation was shown in U87MG cells following 8 h of treatment with ISA27.

MDM2 inhibition is hypothesised to stabilise p53 protein levels by reducing MDM2-mediated p53 degradation. However, p53 stability could also be the result of enhanced transcription of the p53 gene and subsequent p53 protein translation. To confirm that the accumulation of p53 was due to the decreased interaction of p53 with MDM2 rather than elevated p53 expression, we treated GBM cells with ISA27 and monitored the levels of p53 mRNA, p53 protein in the presence of the protein synthesis inhibitor, cycloheximide (CHX), and the MDM2-p53 complex. The transcription of p53 gene did not change in the GBM cells within 12 h of treatment (Fig. 3 A and B), and p53 protein levels persisted even in the presence of CHX (Fig. 3C). Specifically, the inhibition of protein synthesis upon treatment with CHX alone led to a marked decrease in p53 protein expression over time. In ISA27-treated cells, blocking protein synthesis led to an overall increase in p53 protein expression (Fig. 3C). To test the ability of ISA27 to disrupt the intracellular MDM2-p53 interaction, a co-immunoprecipitation assay was done. Only minimal amounts of p53 could be detected in MDM2 immunoprecipitates after 8 h of treatment in GBM cells (Fig. 3D). All together these results indicate that ISA27 is able to induce p53 up-regulation by blocking MDM2-p53 interaction.

**Figure 4:**
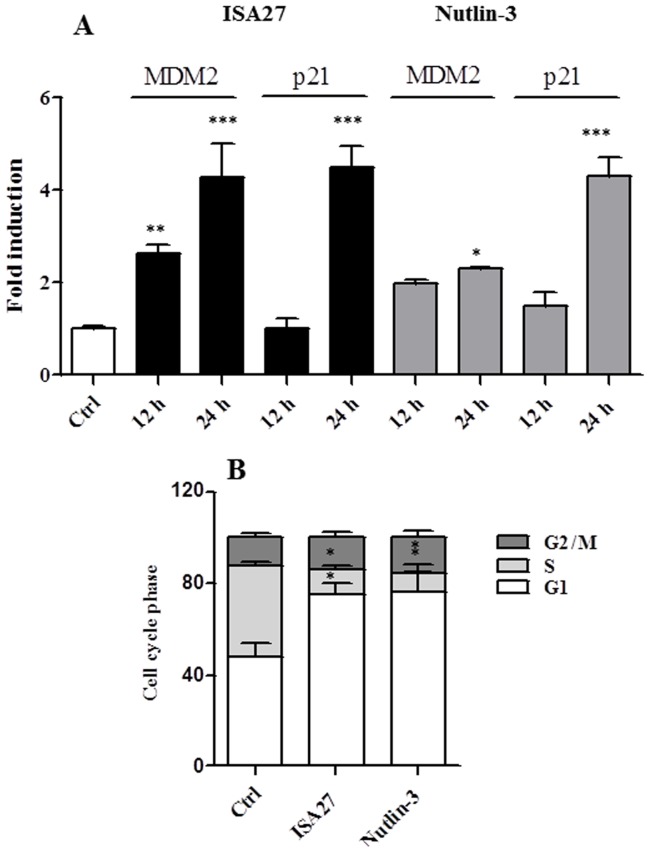
ISA27 restores p53 function in GBM cells. *4A*) *ISA27 induces p53 target gene expression*: Real-time PCR analyses showed a statistically significant increase in MDM2 and p21 mRNA levels following short-term ISA27 treatment in U87MG cells. *4B) ISA27 induces cell cycle arrest*: Cell cycle analysis (24 h) revealed a statistically significant increase in the G1 fraction and a nearly complete depletion of the S-phase population in short-term-treated U87MG cells.

Short-term ISA27 treatment of U87MG cells led to a statistically significant increase in the mRNA levels of p53 target genes; specifically, a 4.3- and 4.5-fold induction of MDM2 and p21 mRNA, respectively, was observed (Fig. 4A). These results suggested that stabilisation of p53 in ISA27-treated GBM cells led to an increase in MDM2 and p21 mRNA levels in a manner that was consistent with the activation of the p53 pathway. Nutlin-3 treatment resulted in a 2.3- and 4.3-fold induction of MDM2 and p21 mRNA, respectively, in U87MG cells (Fig. 4A).

One of the main cellular consequences of p53 activation in proliferating cells is cell cycle arrest at the G1/G2-phase, and the cyclin-dependent kinase inhibitor p21 plays a major role in this arrest. In accord with these data, cell cycle analysis by cytofluorimetric DNA content analysis revealed an increase in the G1 fraction and a nearly complete depletion of S-phase cells after short-term treatment with ISA27 or Nutlin-3 (Fig. 4B).

### 3. Long-term ISA27 Treatment Leads to the Complete Inhibition of Cell Survival/growth in U87MG GBM Cells, but not in Normal Human Lymphomonocytes

**Figure 5:**
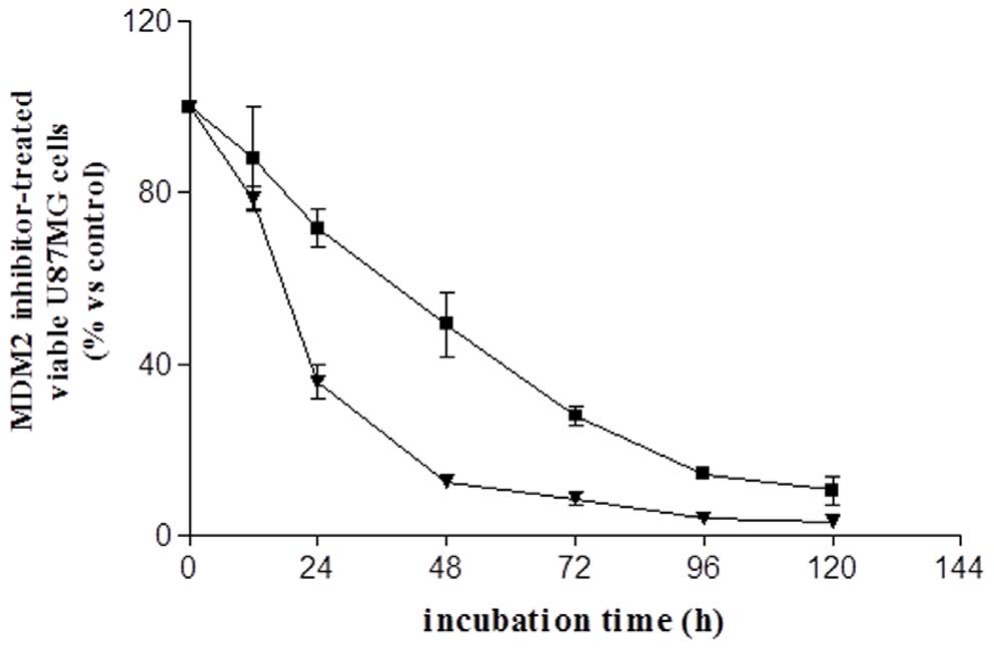
Long-term ISA27 treatment of U87MG cells. U87MG cells were treated with 2.5 µM ISA27 or 10 µM Nutlin-3 for 24, 48, 72, 96 and 120 h. The figure shows the viable cells that were counted by Trypan blue exclusion at the indicated times. The percentage of ISA27- (**▾**) or Nutlin-3- (**▪**) treated viable cells was calculated with respect to untreated viable cells at the indicated incubation times. Points, means of three independent experiments performed in duplicate; bars, SEM.

A substantial decrease in U87MG cell survival/growth was observed after one day of treatment, and the decrease continued at each subsequent time point until almost all viable cells were depleted after 5 days (Fig. 5).

**Figure 6:**
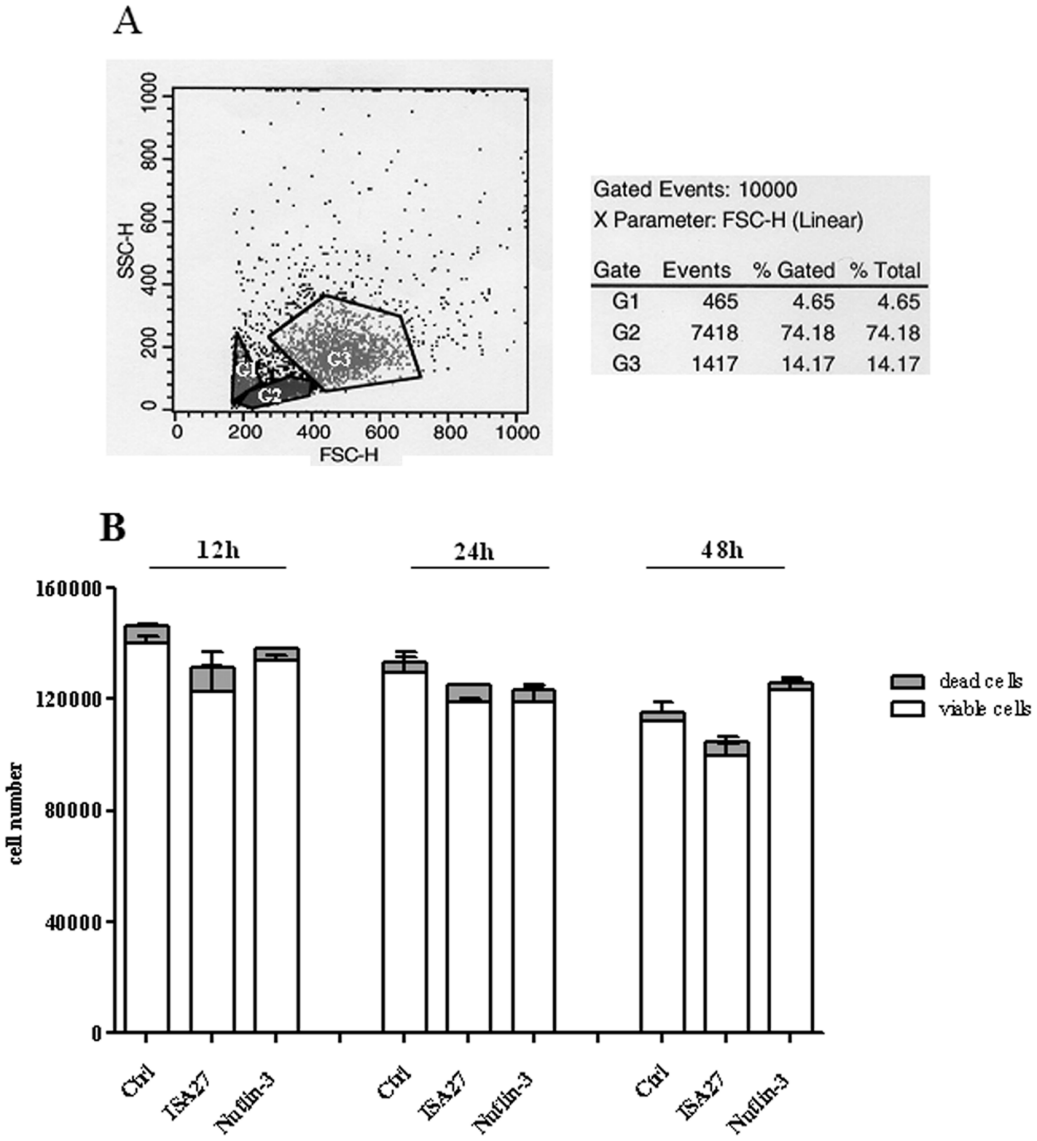
Lack of ISA27 toxicity in an *in vitro* human cell model (lymphomonocytes). *6A*) *Scatter cytogram analysis of cell populations from peripheral blood:* Scatter cytogram analysis showing cell separation by size and granularity (G2 = lymphocytes; G3 = monocytes). One representative experiment is presented. *6B*) *Time-response of human lymphomonocyte viability following ISA27 treatment:* lymphomonocytes were treated with 2.5 µM ISA27 or 10 µM Nutlin-3 for 12, 24 and 48 h. The figure shows the viable cells and dead cells counted by Trypan blue exclusion at the indicated times. Data represent the means of three independent experiments performed in duplicate; bars, SEM. No significant differences were observed between MDM2 inhibitor-treated and untreated-control viable or dead cells at each incubation time.

We also examined the effect of ISA27 on the viability of normal human lymphomonocytes. Flow cytometry was performed to evaluate cell populations isolated from blood samples of healthy individuals. As shown in the scatter cytogram in Fig. 6A, the two cell populations were clearly visible (G2 = 74.0% lymphocytes; G3 = 14.2% monocytes). As shown in Fig. 6B, ISA27-treated lymphomonocytes were still viable at 48 h. Nutlin-3 treatment showed a similar lack of cytotoxicity in normal peripheral blood cells (Fig. 6B).

### 4. Long-term ISA27 Treatment Leads to Permanent Cell Cycle Arrest and Apoptosis in U87MG GBM Cells

**Figure 7:**
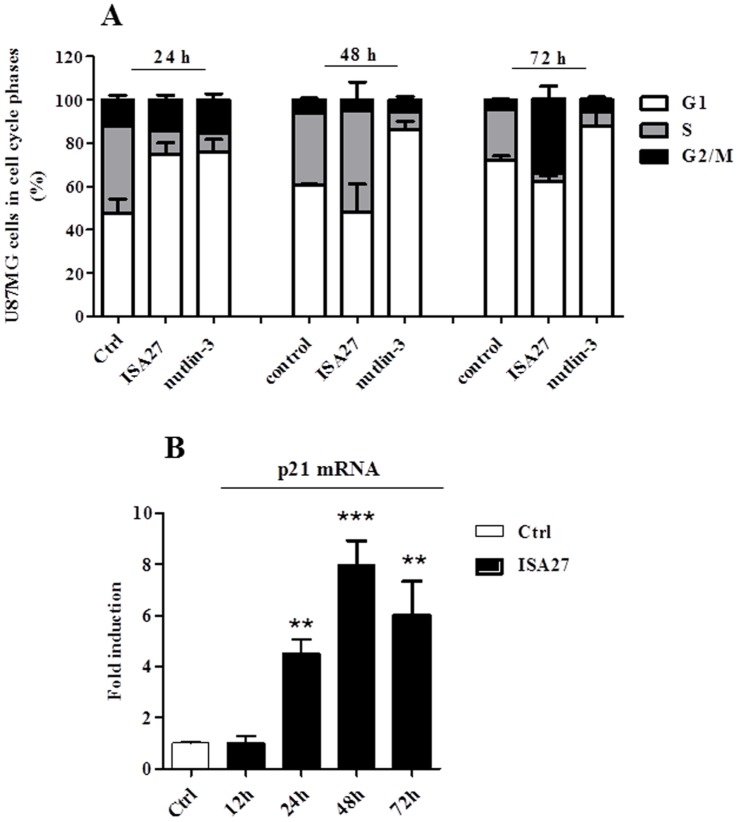
Long-term ISA27 treatment induces cell cycle arrest and a persistent increase in p21 mRNA levels in U87MG cells. *7A) Flow cytometric cell cycle profiling*: the figure shows the percentage of untreated and MDM2 inhibitor-treated U87MG cells in G1-, S- and G2/M-phase. *7B) p21 mRNA evaluation*: ISA27 treatment resulted in a statistically significant increase in p21 mRNA levels at 24, 48 and 72 h.

First, we analysed cell cycle profiling and p21 mRNA levels after long-term ISA27 treatment. ISA27 treatment of U87MG cells for 24 h effectively arrested cell-cycle progression, depleted the S-phase population (p<0.05) and increased the G0/G1-phase population (p<0.05). Treatment for 72 h depleted the S-phase population and increased the G2-phase population (p<0.01) (Fig. 7A). A persistent induction of p21 mRNA was observed, suggesting that the major transcription target of activated p53 was involved in the cell cycle arrest (Fig. 7B). Nutlin-3 arrested cell-cycle progression in U87MG cells 24 and 72 h after treatment, depleting the S-phase compartment and increasing the G0/G1-phase compartment (Fig. 7A).

**Figure 8:**
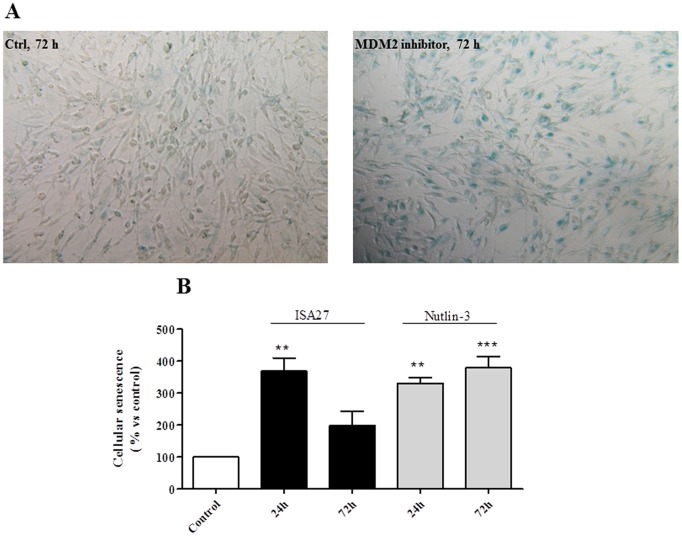
ISA27 induces U87MG cell senescence. *8A*) *Representative images of SA-β-Gal-expressing cells:* The panel shows the SA-β-Gal-expressing MDM2 inhibitor-treated and untreated cells at 72 h. *8B) Percentage of SA-β-Gal-expressing cells:* The number of SA-β-Gal-expressing cells was determined with respect to the total cells in each sample (untreated cells, ISA27- and Nutlin-3-treated cells). The percentage of SA-β-Gal-expressing MDM2 inhibitor-treated cells was then calculated with respect to the untreated cells, for which an arbitrary value of 100% was assigned.

To elucidate whether ISA27 induced permanent cell cycle arrest (cellular senescence), we evaluated whether ISA27 promoted a morphological change in U87MG cells (i.e., large and flat cells) that was compatible with senescence and whether the widely used senescence marker, SA-β-Gal, could be detected in ISA27-treated cells. The ISA27-treated cells acquired an enlarged and flat morphology as revealed by the mean cell diameter (12.36±1.56 µm and 13.59±1.74 µm for control and ISA27-treated cells, respectively). The onset of cellular senescence was rapid; indeed, a high number of U87MG cells were positive for SA-β-Gal after 1 day of treatment. Fig. 8A shows representative images of SA-β-Gal detection in ISA27-treated and control cells. As shown in Fig. 8B, ISA27 significantly enhanced the number of SA-β-Gal-expressing cells after 24 h. Nutlin-3 caused an increase in the number of SA-β-Gal-expressing cells at both 24 and 72 h.

**Figure 9:**
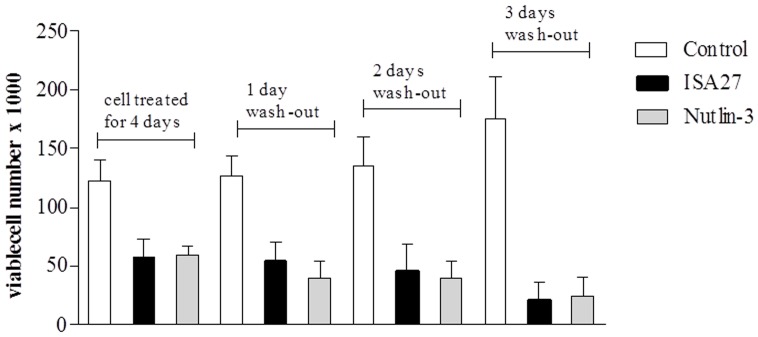
U87MG cells did not recover normal growth upon removal of ISA27. U87MG cells were cultured in the presence of 2.5 µM ISA27 or 10 µM Nutlin-3. After 4 days of MDM2 inhibitor treatment, cell culture medium was replaced with fresh culture medium without the MDM2 inhibitor. The figure shows the number of viable cells that were counted by Trypan blue exclusion after 4 days of MDM2 inhibitor treatment and 1, 2 and 3 days after MDM2 inhibitor removal. Data represent the means of three independent experiments performed in duplicate; bars, SEM.

Next, to assess whether U87MG cells could resume growing after ISA27 removal, culture medium was replaced with fresh medium not containing ISA27 after 4 days of cell treatment, and the viable cells were assessed after 1, 2 and 3 days. After ISA27 removal, the number of viable cells further decreased at these time points with respect to control samples, suggesting the inability to recover normal cell growth (Fig. 9).

To investigate whether ISA27 induced apoptosis, the following parameters were evaluated: dissipation of mitochondrial potential (Δ*Ψ*m), cytosolic cytochrome c content, PUMA mRNA levels and DNA fragmentation.

**Figure 10:**
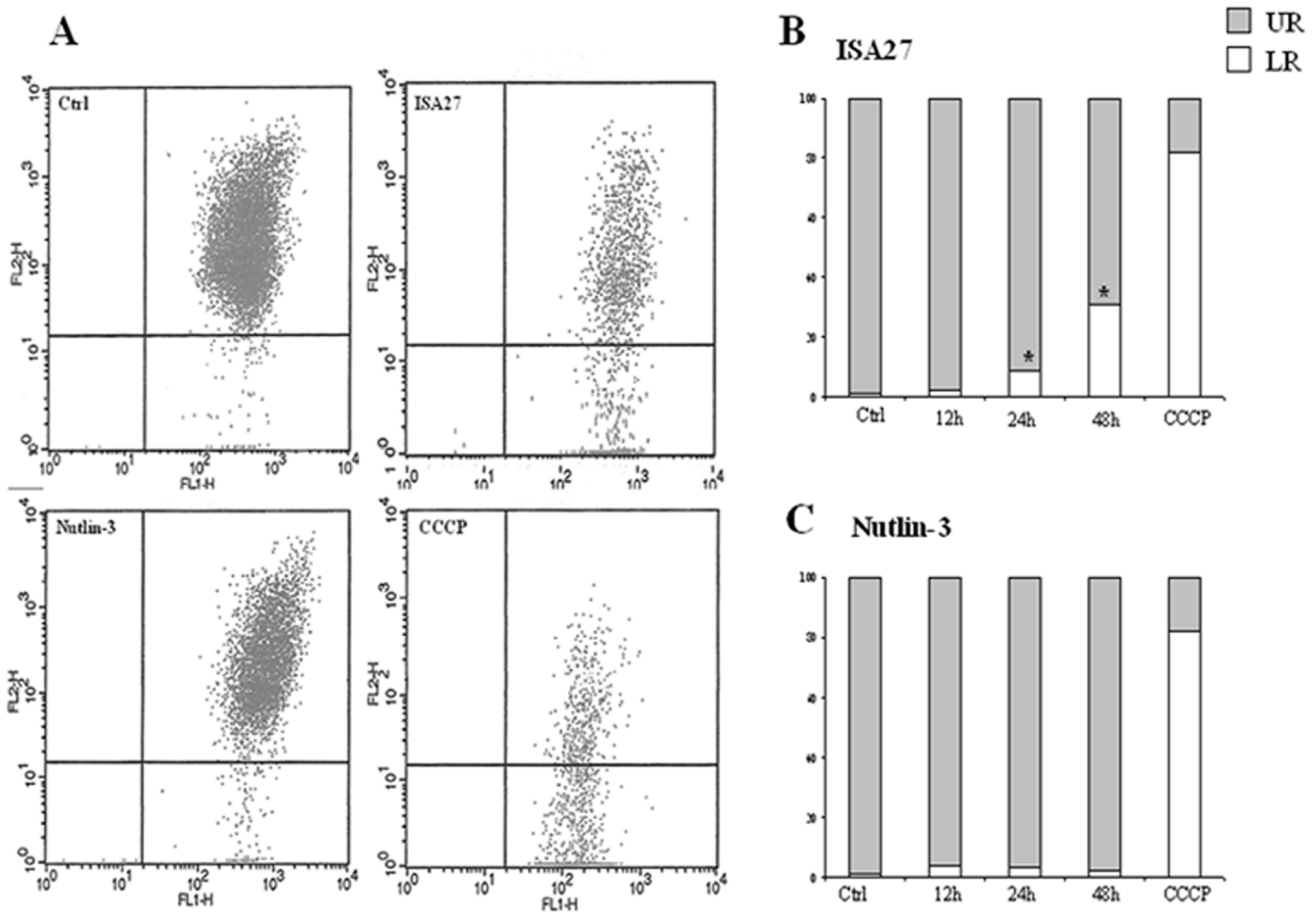
ISA27 induces the dissipation of the mitochondrial membrane potential. *10A) Representative dot plots of untreated and MDM2 inhibitor-treated cells:* After ISA27 treatment, mitochondrial depolarisation is visible by a decrease and an increase in fluorescence in the FL-2 and FL-1 channels, respectively. After Nutlin-3 treatment, mitochondrial depolarisation is not visible; CCCP is the positive control. *10B and C) Time-course analysis of ΔΨm dissipation:* Histograms show the mean values of cell percentages either in the UR (polarised mitochondria) or LR (depolarised mitochondria) quadrant of the Δ*Ψ*m analysis plots derived from three independent experiments.

A decrease in Δ*Ψ*m was indicated by a reduction in orange JC-1 aggregate fluorescence (recorded by the FL2 channel of the flow cytometer) accompanied by a concomitant increase in green JC-1 monomer fluorescence (recorded by the FL1 channel). Representative examples of the flow cytometric analysis are shown in Fig. 10A. The majority of untreated cells (99%) showed high fluorescence emission in both channels and were found in the upper right (UR) quadrant of the plot. The remaining untreated cells (1%) showed low fluorescence emission in FL2 and were therefore found in the lower right (LR) quadrant. Upon ISA27 treatment, an increase was observed in the percentage of the cells plotting in the LR quadrant, consistent with Δ*Ψ*m dissipation. In particular, significant changes in Δ*Ψ*m were observed after treatment for 24 and 48 h (8.5% and 31%, respectively; P<0.05) as shown in Fig. 10B.

**Figure 11:**
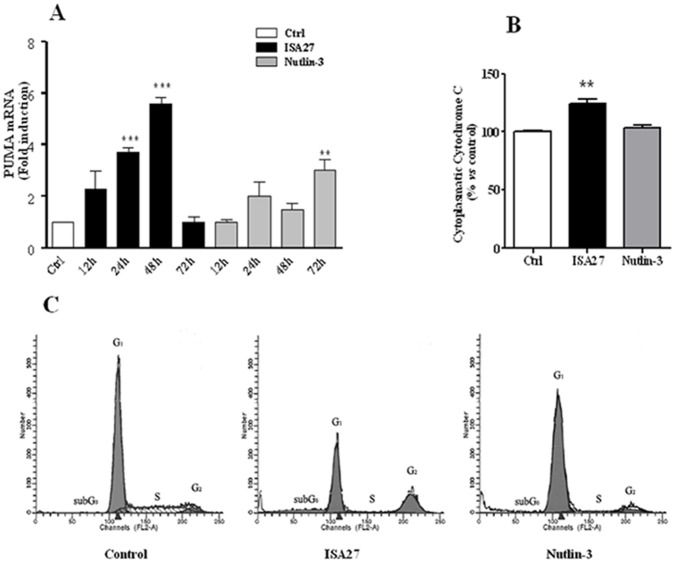
ISA27 induces an increase in PUMA mRNA levels, mitochondrial cytochrome c release, and DNA fragmentation. *11A) Relative quantification of PUMA mRNA:* ISA27 induced a statistically significant increase in PUMA mRNA levels at 24 and 48 h. Nutlin-3 treatment resulted in a statistically significant increase at 72 h. *11B) Evaluation of cytosolic cytochrome c content*: ISA27-treated cells showed a 25% increase in cytochrome c levels in the cytosolic fraction. Nutlin-3-treated cells did not give statistically significant results. *11C) Evaluation of DNA content*: Frequency histograms from a representative experiment are shown. ISA27-treated cells showed a significant increase in the percentage of nuclei with hypodiploid DNA content at 72 h compared with control cells. In contrast, Nutlin-3 did not induce significant nuclear DNA fragmentation.

Real time RT-PCR analysis showed that ISA27 treatment led to a statistically significant increase in PUMA mRNA at 24 and 48 h compared with the control (Fig. 11A).

Figure 11B shows that ISA27-treated cells exhibited a 25% increase in cytochrome c in the cytosolic fraction compared with the control.

DNA-specific PI staining showed that treatment with ISA27 caused a significant increase in the percentage of cells with hypodiploid DNA content, a clear sign of apoptosis, as shown in the representative frequency histograms (Fig. 11C). In particular, the percentage of apoptotic cells (sub-G0 cells) observed after exposure of U87MG cells to ISA27 for 72 h increased to 17.7±1.27% (P<0.001), whereas that of the control was 0.43±0.05%. Nutlin-3 treatment did not give statistically significant results after the indicated incubation times for these apoptotic parameters (Fig. 11B and C) with the exception of increased PUMA mRNA levels after treatment for 72 h (Fig. 11A).

**Figure 12:**
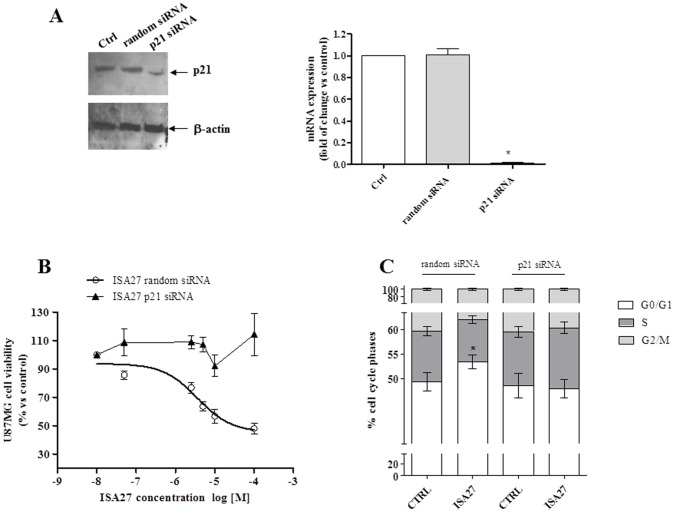
siRNA against p21 abrogates ISA27-mediated cell growth inhibition. A) *Evaluation of p21 levels after transient transfection of U87MG cells with p21 siRNA:* Western blotting and real-time RT-PCR analyses demonstrated decreased p21 levels in U87MG cells after 24 h transient transfection. *B) Evaluation of ISA27 effect on cell viability.* p21 siRNA samples were exposed to a range of ISA27 concentrations for 24 h. After incubation, U87MG cell viability was determined by MTS assay. The IC50 value was 3.6±0.5 µM for random siRNA ISA27 sample. Transfection of U87MG cells with p21 siRNA rendered ISA27 much less effective to inhibit cell viability. *C) Evaluation of ISA27 effect on cell cycle in p21 siRNA sample:* random siRNA and p21siRNA samples were exposed to a fixed dose of ISA27 (5 µM) or DMSO for 24 h. The ISA27-treated random siRNA sample showed the increase of G1 phase compared to random siRNA sample. The comparison of cell cycle phases between p21 siRNA and ISA27-treated p21 siRNA sample did not show statistically significant differences.

**Figure 13:**
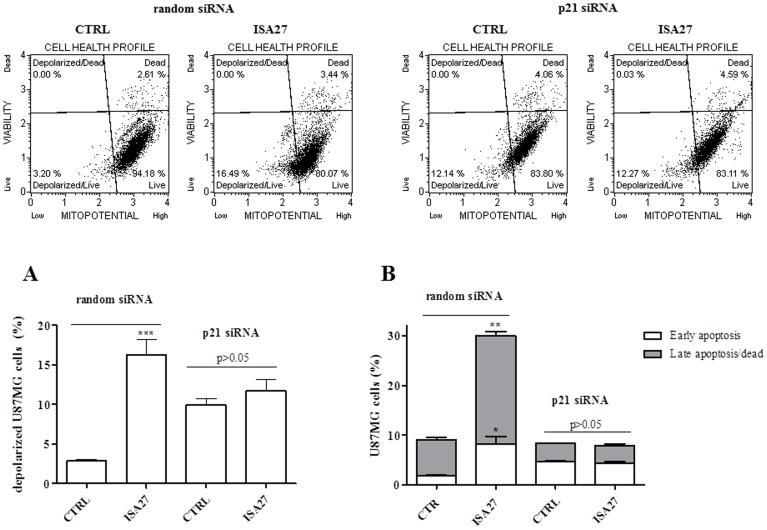
siRNA against p21 made ISA27 unable to induce ΔΨm dissipation and PE. Random siRNA and p21siRNA samples were exposed to a fixed dose of ISA27 (5 µM) or DMSO for 24 h. A) *Evaluation of ISA27 effect on the ΔΨm in p21 siRNA samples*. The ISA27-treated random siRNA sample showed dissipation of ΔΨm compared to random siRNA sample. The comparison of ΔΨm between p21 siRNA and ISA27-treated p21 siRNA sample did not show statistically significant differences. Upper panel shows representative dot plots of untreated and ISA27-treated random or p21siRNA samples. *B) Evaluation of ISA27 effect on PE in p21 siRNA samples.* The ISA27-treated random siRNA sample showed a statistical significant increase of early and late apoptosis compared to random siRNA sample. The comparison of early and late apoptosis between p21 siRNA and ISA27-treated p21 siRNA sample did not show statistically significant differences.

To explore the role of p21 in the ISA27-induced cell effects, we analysed cell viability, cell cycle profile and apoptosis markers in ISA27-treated U87MG cells in which p21 was genetically inhibited. First, to verify p21 down-regulation by siRNA, the levels of p21 mRNA and protein were evaluated in U87MG cells transfected with p21 siRNA after 24 and 48 h. The Fig. 12 shows that p21 protein (Fig. 12A) and mRNA (Fig. 12B) were effectively down-regulated post 24 h p21 siRNA transfection. For subsequent experiments, we down-regulated p21 and incubated U87MG cells with ISA27 for 24 h. In p21 siRNA sample, ISA27 failed to reduce U87MG cell viability (Fig. 12 B) and arrest cell cycle (Fig. 12 C). Furthermore, ΔΨm flow cytometry and Annexin V FITC analysis showed that p21 down-regulation made ISA27 unable to effectively induce mitochondrial potential dissipation (Fig. 13A) and phosphatidylserine externalization (PE) (Fig. 13B) in U87MG cells.

### 5. Effects of ISA27 on GBM Cell Growth in vivo

**Figure 14:**
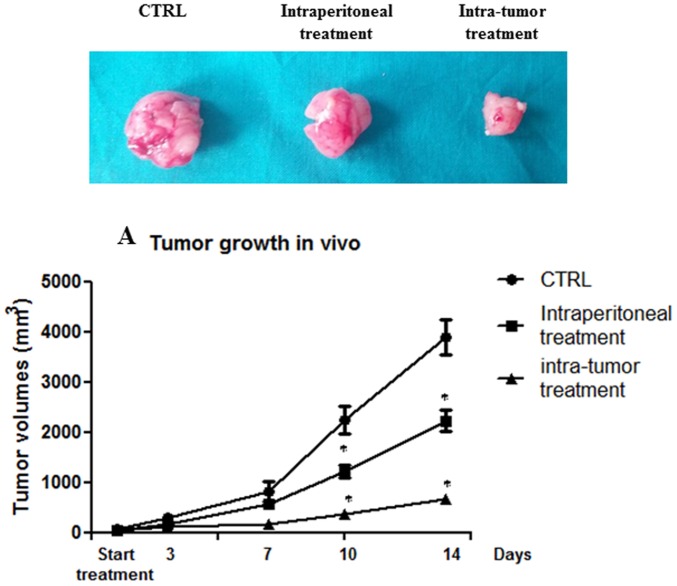
*In vivo* effects of ISA27 on GBM growth. After inoculation of U87MG cells into BALB/c nude mice, tumor growth was measured twice a week (for 14 days of treatment) with a caliper. Both intra-peritoneal (5 mg/kg) and intra-tumoral (3 mg/kg) treatments with ISA27 delayed tumor growth compared with controls. As shown in the graph, the intra-tumoral treatment was more effective for inhibition of tumor growth. The figure also shows a representative image of tumors at the end of the treatment. All values are presented as the mean of the values observed in mice from the same group (5 mice/group). Two-way ANOVA was performed to compare the different parameters among the different groups. A significance level of *P*<0.05 was assumed for all statistical evaluations. Statistics were computed using GraphPad Prism software.

**Figure 15:**
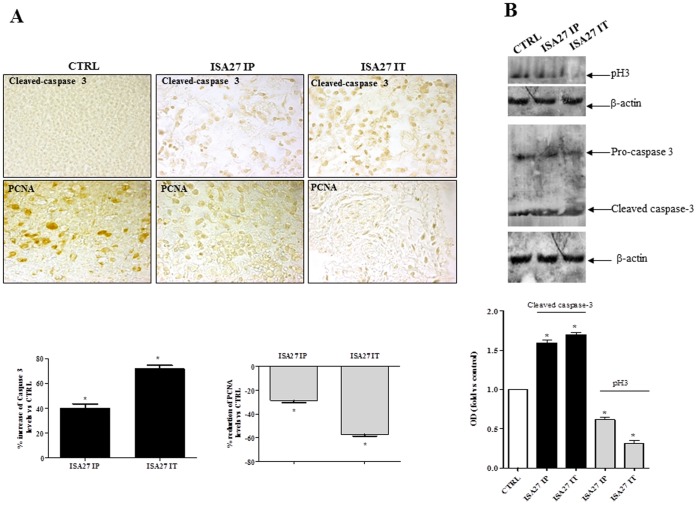
ISA27 inhibits cell proliferation and induces apoptosis in tumor tissues. 14 days from starting treatment, tumors were harvested from mice of different ISA27 treatment groups (IP = intraperitoneal treatment; IT = intratumor treatment) and processed for histological and Western blotting analysis. *A) Histological analysis:* apoptosis and cell proliferation were evaluated by analysis of cleaved caspase 3 and PCNA levels by immunohistochemistry in paraffin embedded sections of tumors. Upper panel shows representative images of immunohistochemistry analysis. ISA27-treated tumors show increased cleaved caspase 3 levels and reduced cell proliferation. Cleaved caspase 3 and PCNA levels in tumors were quantified from digital images. Results are shown in graph as percent of cleaved caspase 3 and PCNA expression respect to controls (*P<0.05 *vs* control). *B) Western blotting analysis:* apoptosis and cell proliferation were evaluated by analysis of cleaved caspase 3 and phosphorylated histone H3 (pH3) levels by Western blotting in whole lysates of tumors using specific antibodies. Densitometric analysis shows that ISA27 induced a significant increase in cleaved-caspase-3 levels and a significant reduction in pH3 levels (*P<0.05 *vs* control).

**Figure 16:**
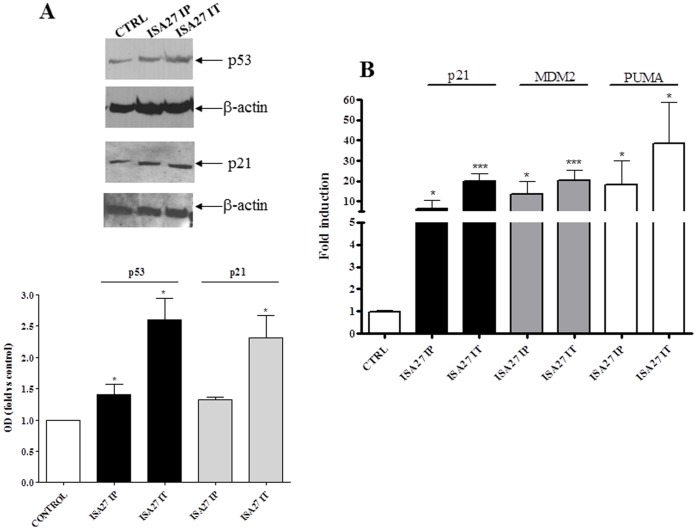
ISA27 activates p53 *in vivo*. Tumors were harvested from mice of different ISA27 treatment groups (IP = intraperitoneal treatment; IT = intratumor treatment), processed to obtain cell lysates for Western blot and total RNA for real-time RT-PCR analysis. *A)* Western blot analysis showed up-regulation of p53 and p53 transcriptional target p21 in tumors from ISA27 IT group and up-regulation of p53 in tumors from ISA27 IP group. In this group, the increase of p21 was near to significance. *B)* Real-time RT-PCR analysis showed a statistically significant increase in MDM2, p21 and PUMA mRNA levels in tumors from ISA27 IT and IP groups.

In nude mice, the injection of 2×10^6^ U87MG cells into the dorsal lateral region resulted in the development of an ∼6 mm-diameter tumor in 2 weeks in approximately 70% of the mice. Tumors were treated with an intra-peritoneal and intra-tumoral injection of ISA27. After 14 days of treatment, the mice were sacrificed, and internal organs were removed for histological analysis. Fig. 14 shows that both intra-peritoneal and intra-tumoral treatments retarded tumor growth (Fig. 14). The *in vivo* treatment with ISA27 inhibited cell proliferation and induced apoptosis in tumor tissues. Specifically, the immunohistochemical and Western blot analysis showed that the injection of ISA27 increased levels of the apoptosis marker cleaved-caspase-3 and reduced levels of the proliferation markers PCNA and phosphorylated histone H3 (Fig. 15A and B). To show that the antitumor effect of ISA27 is caused by activation of the p53 pathway we analysed the level of p53 and p53-regulated genes in ISA27-treated U87MG xenografts. Western blot analysis revealed accumulation of p53 that was accompanied by up-regulation of p21 in tumors from ISA27-treated mice but not vehicle controls (Fig. 16A). Real-time RT-PCR data confirmed these findings and further showed that p53 accumulation induced by ISA27 was accompanied by up-regulation of MDM2, p21 and PUMA mRNA in tumor tissues (Fig. 16B). These experiments are consistent with the hypothesis that the *in vivo* antitumor effect of ISA27 is derived from activation of the p53 pathway.

**Figure 17:**
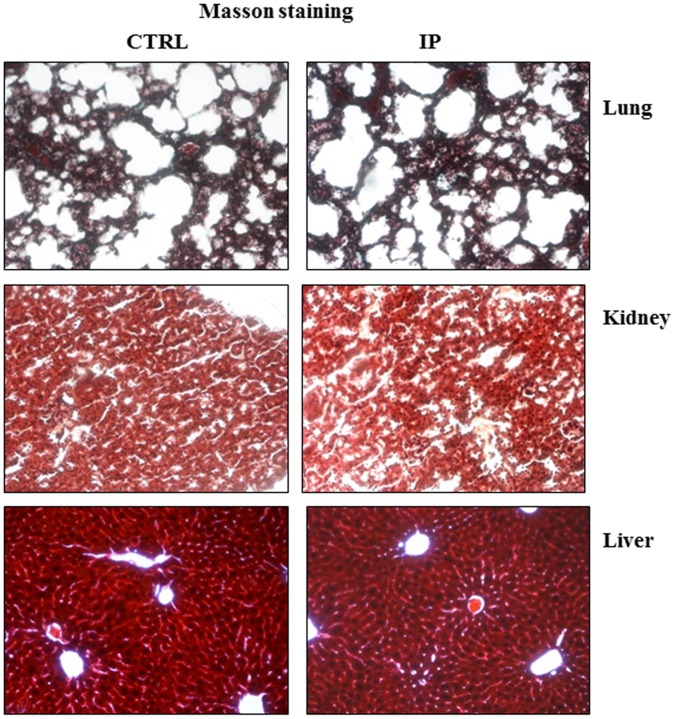
ISA27 is not toxic to normal tissues. Two weeks from the start of the treatment, mice were sacrificed, and internal organs were removed for histological analysis. Masson trichrome staining was performed on liver, kidney and lung tissues from treated and control mice. No morphological differences were found in the treated mice compared with the controls.

Finally, we tested the safety of the *in vivo* administration of ISA27 in healthy nude mice. There were no significant changes in body weight among treated and control groups of mice, indicating no toxicity of the treatment (data not shown). Internal organs (liver, lung, kidney) were then analysed by histology. No morphological differences were found in treated mice compared with controls (Fig. 17).

### 6. Synergism between ISA27 and the Conventional Chemotherapeutic Drug, Temozolomide

**Figure 18:**
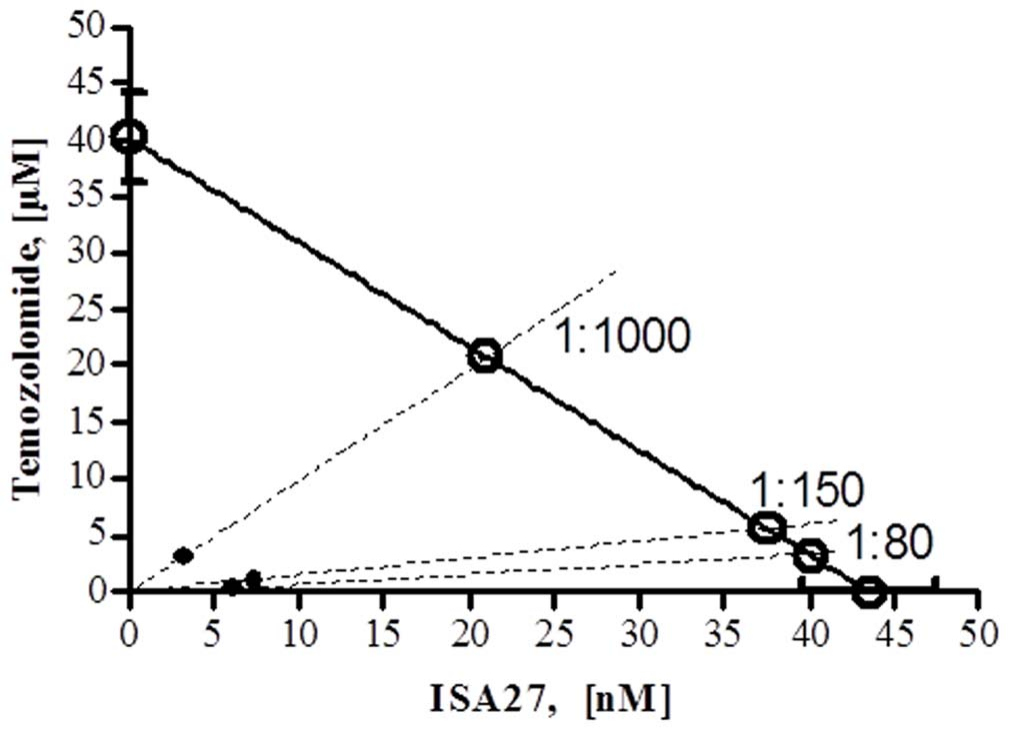
Synergistic effect of ISA27 and temozolomide on the survival/growth of GBM cells. Isobologram 2-D showing the interactions between TMZ and ISA27 in MTS viability tests performed in U87MG cells treated for 72 h with TMZ and/or ISA27. The IC_50_ values for TMZ and ISA27 are shown on the X- and Y-axes, respectively. The isobole of additivity is shown as a solid line drawn between the aforementioned IC_50_ values of TMZ and ISA27 and connects the X- and Y-axes. The open points (○) on the additivity line depict the theoretical IC_50,add_ values for total dose expressed as the proportion of TMZ and ISA27 that produced a 50% effect. The solid points (•) depict the experimental IC_50,mix_ values for total dose expressed as the proportion of TMZ and ISA27 that produced a 50% effect. The experimental IC_50,mix_ values of the mixture of ISA27 and TMZ for the fixed-ratio combinations of 1∶80, 1∶150 and 1∶1,000 were found to be significantly below the theoretical isoboles of additivity, indicating super-additive (synergy) interactions.

Because both MDM2 inhibitors and genotoxic drugs use the p53 pathway, they may synergise to promote cytotoxic activity in GBM cells that retain p53 function. To this end, we investigated the effect of combining doses of ISA27 with the currently used genotoxic drug, temozolomide (TMZ). Based on the IC_50_ values for ISA27 and TMZ alone, the theoretical additive IC_50,add_ values for ISA27 and TMZ were calculated for three fixed ratios (1∶80, 1∶150, 1∶1,000) (Fig. 18). Furthermore, the experimental IC_50,mix_ values were determined for the same fixed-ratio combinations in the viability assay (Fig. 13). The IC_50,add_ and the IC_50,mix_ values are shown in Table 2. Statistical analysis of the data from isobolographic analysis revealed synergistic interactions between ISA27 and TMZ for the three examined fixed-ratio combinations (Table 2; Fig. 18). Furthermore, the interaction index values of the combinations demonstrated the following rank of potencies: 1∶80>1∶1,000>1∶150 (ISA27:TMZ).

**Table 2:** Effect of ISA27, Temozolomide (TMZ) and their fixed-ratio combinations with regard to growth inhibition of U87MG cells.

ISA27/TMZ combinations	IC_50,mix_ (µM)	IC_50,add_ (µM)	γ
**ISA27 alone**	0.044±0.001	**–**	**–**
**TMZ alone**	40.21±4.73	**–**	**–**
**1∶80**	3.25±0.24	1.39±0.42	0.306
**1∶150**	5.67±0.48	1.88±0.34	0.390
**1∶1000**	20.93±2.87*	7.78±0.69	0.314

Data are presented as IC_50_±S.E.M. Statistical analysis was performed with Student’s *t*-test.

* P<0.05 *vs* the respective additive group. γ <1 indicates supra-additivity (synergy).

## Discussion

The direct and specific activation of the p53 pathway without inducing collateral DNA damage offers a tantalising solution to the shortcomings of current therapeutic regimens and appears to be a reasonable approach for GBM therapy in view of the infrequent occurrence of p53 gene mutations [9], [10]. The cumulative evidence of aberrantly increased activity of the primary p53 inhibitor MDM2 in GBM [11], [12] prompted us to examine the effects of targeted inhibition of the MDM2-p53 interaction by the spiro-oxindole analogue ISA27, a recently described small-molecule inhibitor of MDM2 [26]. Little is known about the effects of MDM2 inhibitors on the *in vitro* growth of GBM cells. Recently, Nutlin-3, the first potent small-molecule inhibitor of MDM2 [23], was reported to be effective at inhibiting GBM cell growth *in vitro* [25], suggesting the validity of this experimental approach for the treatment of GBM.

In this study, we investigated whether ISA27 affected the growth of GBM cells and explored the intracellular events following ISA27 treatment. The U87MG and U343MG cell lines overexpress MDM2 and maintain wild-type p53 [43]–[45] and were chosen as a cell culture model of human GBM. In these cell lines, the primary mechanism of p53 inactivation is the high nuclear MDM2 levels caused by a lack of PTEN, a tumor suppressor protein that normally counteracts MDM2 translocation into the nucleus [46]. The lack of PTEN makes these cell lines a suitable representative model of GBM, as the loss of the PTEN gene locus has been found in up to 80% of GBM cases [9], [10], [13].

This is the first report to demonstrate that ISA27 is a potent inhibitor of GBM cell growth. Previous studies have shown that ISA27 activates p53, resulting in growth inhibition in HEK-293 transformed human embryonic kidney, M14 human melanoma and U937 human monocyte lymphoma cell lines [26]. Our results demonstrate that ISA27 blocks the cell cycle and triggers an apoptotic cell death program in the GBM cells, responses that are similar to those obtained in human M14 melanoma cells.

We observed a dose-dependent antiproliferative effect of ISA27 in U87MG and U343 cells following short-term treatments (24 h) with increasing ISA27 concentrations (the IC_50_ values of ISA27 were 2.5±0.4 µM and 5.7±0.9 µM in U87MG and U343 MG cells, respectively). The specificity of the antiproliferative effects was demonstrated by the accumulation of the p53 protein due to the decreased interaction between p53 and MDM2 and the restoration of p53 function in GBM cells after ISA27 treatment. The reactivation of p53 was suggested by the transcriptional activation of the primary target genes of p53, *MDM2* and *CDKN1A*. The *CDKN1A* gene encodes the cyclin-dependent kinase inhibitor p21, an essential mediator of p53-induced cell cycle arrest. Exponential growth of U87MG cells was significantly inhibited following long-term ISA27 treatment. The number of viable cells was substantially reduced after 1 day of ISA27 treatment, and this reduction reached almost total growth inhibition after 5 days. Kinetic analyses of the ISA27-induced intracellular effects showed that the decrease in viable cells was mainly due to a G1-phase cell cycle block and cellular senescence during the initial phase of ISA27 treatment. However, apoptotic parameters, such as the dissipation of mitochondrial membrane potential, began to appear. Prolonged ISA27 exposure caused a further decline in viable cells due to a G2-phase cell cycle block and apoptosis. Notably, the remaining cells did not recover normal growth following removal of ISA27, suggesting the irreversible impairment of mechanisms regulating cell cycle progression.

By analysing U87MG gene expression and apoptotic parameters in response to ISA27, we identified the upregulation of the *PUMA* gene, which is involved in mediating the apoptotic response of p53, including mitochondrial potential dissipation, cytochrome c release into the cytoplasm and DNA fragmentation, all features that are consistent with apoptotic cell death. The genetic inhibition of p21 using siRNA abrogated the effects of ISA-27 on cell cycle arrest, suggesting a crucial role of p21 in the cell growth inhibition induced by ISA27. Furthermore, the down-regulation of p21 made ISA27 unable to induce significantly mitochondrial potential dissipation and phosphatidylserine externalization, suggesting an important role of p21 in ISA27-mediated effects. To the best of our knowledge, no data are available about molecular p21 involvement in MDM2 inhibitor effects except in p21-downregulated pancreatic cancer cells, in which the block of the apoptotic potential by the MDM2 inhibitor MI-319 has been demonstrated [19].

The *in vitro* antitumor activity of ISA27 was confirmed *in vivo* using GBM U87MG cell xenografts in nude mice. ISA27 treatment of mice bearing tumors 600 mm^3^ in size resulted in approximately 85% inhibition of tumor growth relative to vehicle controls.

It is important to note that the long-term treatment with Nutlin-3 *in vitro* effectively inhibited U87MG cell growth. The primary cellular response to Nutlin-3 was permanent cell cycle arrest that continued until the end of the treatment period. Only in the final stage of treatment did signs of apoptosis begin to appear, as indicated by high levels of PUMA mRNA. This finding is consistent with literature that has reported U87MG cell apoptosis after Nutlin-3 treatment for 96 h [25].

It is not clear how Nutlin-3 and ISA27 stimulate cellular responses with different kinetics in U87MG cells. Cellular responses with different kinetics have been recently shown for ISA27 and another small molecule, 10d, in the M14 human melanoma cell line [26]. Treatment with ISA27 for 24 h induced both cell cycle arrest and apoptosis, whereas 10d caused cell cycle arrest only. In U87MG cells, the ability of ISA27 to promote a cell cycle block in combination with apoptosis could be attributed to the more rapid accumulation of p53 protein levels during treatment with respect to Nutlin-3 treatment. The rapid ISA27-induced increase in p53 protein could find a more favourable cellular environment to efficiently activate p53 function as indicated by the induction of MDM2 and proapoptotic PUMA gene transcription. This rapid ISA27-induced antiproliferative response may be beneficial in the treatment of human GBM, considering that this cancer is characterised by rapid cell growth. Additionally, a lower dose of ISA27 was efficacious when compared with Nutlin-3. The implication of this result can be illustrated from the recent Phase I study that showed the clinical efficacy of the MDM2 inhibitor, JNJ-26854165, in patients with advanced solid tumors, but at elevated doses, some toxic effects were reported [47]. For example, lymphopoenia was observed in the majority of the patients, and more than 20% experienced grade 3 or 4 severity [47]. In this context, the ability of ISA27 to maintain the viability of human lymphomonocytes is of particular interest. A selective toxic effect of MDM2 inhibitors on cancer cells has been shown by other authors using a number of normal cell models. It has been demonstrated that Nutlin-3 is not toxic to peripheral blood mononuclear cells, bone marrow-derived haematopoietic progenitors and bone marrow stromal epithelial cells [48]–[50]. The administration of ISA27 *in vivo* stimulated p53 activation in the xenograft model of human GBM, resulting in inhibition of cell proliferation and induction of apoptosis. ISA27 showed antitumor activity without causing visible signs of toxicity in the animals as assessed by necroscopy and body weight assessment. These results are in agreement with previous *in vivo* studies performed with Nutlin-3 and other MDM2 inhibitors [51], [52].

The precise mechanism of cell death resistance in normal cells remains unclear. The resistance may be a consequence of the low basal expression levels of the MDM2 oncoprotein in normal cells. Thus, following cell treatment with the MDM2 inhibitor, the amount of p53 protein dissociated from MDM2 and accumulated would not be sufficient to trigger cell death. In contrast, tumor cells overexpress MDM2, which sequesters high amounts of p53. Consequently, after blocking the interaction between these two proteins, the high accumulation of p53 renders the cells highly susceptible to p53 reactivation and more sensitive to apoptosis [52].

From a therapeutic perspective, it is interesting that ISA27 in combination with the conventional chemotherapy drug TMZ inhibited U87MG cell growth. This combination worked in a synergistic manner as confirmed by isobolographic analysis (γ <1 in all cases). This result suggests the possibility of lowering the dose of TMZ used in the treatment of GBM.

In conclusion, our data show that ISA27 disrupts the MDM2-p53 interaction and releases the powerful antitumor capacities of p53 in GBM cells. The use of this MDM2 inhibitor could offer a novel therapy for the treatment of GBM patients by inhibiting tumor growth.

## References

[1] Liu J, Ma Q, Zhang M, Wang X, Zhang D, et al (2012) Alterations of TP53 are associated with a poor outcome for patients with hepatocellular carcinoma: Evidence from a systematic review and meta-analysis. Eur J Cancer 48: 2328–2338. 22459764 10.1016/j.ejca.2012.03.001 PMC3395767

[2] Furnari FB, Fenton T, Bachoo RM, Mukasa A, Stommel JM, et al (2007) Malignant astrocytic glioma: genetics, biology, and paths to treatment. Genes Dev 21: 2683–2710. 17974913 10.1101/gad.1596707

[3] Ohgaki H, Dessen P, Jourde B, Horstmann S, Nishikawa T, et al (2004) Genetic pathways to glioblastoma: a population-based study. Cancer Res 64: 6892–6899. 15466178 10.1158/0008-5472.CAN-04-1337

[4] Conrad CA, Milosavljevic VP, Yung WK (1995) Advances in chemotherapy for brain tumors. Neurol Clin 13: 795–812. 8583997

[5] Levin VA, Leibel SA, Gutin PH (2001) Neoplasms of the central nervous system. In: De Vita Jr VT, Hellman S, Rosenberg SA, editors. Cancer principles of oncology. Philadelphia: Lippincott-Raven. 2100–2161.

[6] Desjardins A, Rich IN, Quinn JA, Vredenburgh J, Gururangan S, et al (2005) Chemotherapy and novel therapeutic approaches in malignant glioma. Front Biosci 10: 2645–2668. 15970525 10.2741/1727

[7] Hess KR, Broglio KR, Bondy ML (2004) Adult glioma incidence trends in the United States, 1977–2000. Cancer 101: 2293–2299. 15476282 10.1002/cncr.20621

[8] Nagasawa DT, Chow F, Yew A, Kim W, Cremer N, et al (2012) Temozolomide and other potential agents for the treatment of glioblastoma multiforme. Neurosurg Clin N Am. 23: 307–322. 10.1016/j.nec.2012.01.007 22440874

[9] Ohgaki H, Kleihues P (2009) Genetic alterations and signaling pathways in the evolution of gliomas. Cancer Sci 100: 2235–2241. 19737147 10.1111/j.1349-7006.2009.01308.x PMC11159448

[10] Cerami E, Demir E, Schultz N, Taylor BS, Sander C (2010) Automated network analysis identifies core pathways in glioblastoma. PLoS One 5: e8918. 20169195 10.1371/journal.pone.0008918 PMC2820542

[11] He J, Reifenberger G, Liu L, Collins VP, James CD (1994) Analysis of glioma cell lines for amplification and overexpression of MDM2. Genes Chrom Cancer 11: 91–96. 7529554 10.1002/gcc.2870110205

[12] Freedman DA, Wu L, Levine AJ (1999) Functions of the MDM2 oncoprotein. Cell Mol Life Sci 55: 96–107. 10065155 10.1007/s000180050273 PMC11146946

[13] Halatsch ME, Schmidt U, Unterberg A, Vougioukas VI (2006) Uniform MDM2 overexpression in a panel of glioblastoma multiforme cell lines with divergent EGFR and p53 expression status. Anticancer Res 26: 4191–4194. 17201132

[14] Li C, Shen J, Wei X, Xie C, Lu W (2012) Targeted delivery of a novel palmitylated D-peptide for antiglioblastoma molecular therapy. J Drug Target 20: 264–271. 22233211 10.3109/1061186X.2011.645162

[15] Wang B, Fang L, Zhao H, Xiang T, Wang D (2012) MDM2 inhibitor Nutlin-3a suppresses proliferation and promotes apoptosis in osteosarcoma cells. Acta Biochim Biophys Sin (Shanghai). 44: 685–691. 10.1093/abbs/gms053 22843172

[16] Henze J, Mühlenberg T, Simon S, Grabellus F, Rubin B, et al (2012) p53 modulation as a therapeutic strategy in gastrointestinal stromal tumors. PLoS One 7: e37776. 22662219 10.1371/journal.pone.0037776 PMC3360609

[17] Shangary S, Wang S (2009) Small-molecule inhibitors of the MDM2-p53 protein-protein interaction to reactivate p53 function: a novel approach for cancer therapy. Annu Rev Pharmacol Toxicol 49: 223–241. 18834305 10.1146/annurev.pharmtox.48.113006.094723 PMC2676449

[18] Vu BT, Vassilev L (2011) Small-molecule inhibitors of the p53-MDM2 interaction. Curr Top Microbiol Immunol 348: 151–172. 21046355 10.1007/82_2010_110

[19] Azmi AS, Aboukameel A, Banerjee S, Wang Z, Mohammad M, et al (2010) MDM2 inhibitor MI-319 in combination with cisplatin is an effective treatment for pancreatic cancer independent of p53 function. Eur J Cancer 46: 1122–1131. 20156675 10.1016/j.ejca.2010.01.015 PMC4106027

[20] Sonnemann J, Palani CD, Wittig S, Becker S, Eichhorn F, et al (2011) Anticancer effects of the p53 activator nutlin-3 in Ewing's sarcoma cells. Eur J Cancer 47: 1432–1441. 21334198 10.1016/j.ejca.2011.01.015

[21] Ghassemifar S, Mendrysa SM (2012) MDM2 antagonism by nutlin-3 induces death in human medulloblastoma cells. Neurosci Lett. 513: 106–110. 10.1016/j.neulet.2012.02.022 22343310

[22] Chappell WH, Lehmann BD, Terrian DM, Abrams SL, Steelman LS, et al (2012) p53 expression controls prostate cancer sensitivity to chemotherapy and the MDM2 inhibitor Nutlin-3. Cell Cycle 11: 4579–4588. 23187804 10.4161/cc.22852 PMC3562303

[23] Vassilev LT, Vu BT, Graves B, Carvajal D, Podlaski F, et al (2004) In vivo activation of the p53 pathway by small-molecule antagonists of MDM2. Science. 303: 844–888. 10.1126/science.1092472 14704432

[24] Liu M, Li C, Pazgier M, Li C, Mao Y, et al (2010) D-peptide inhibitors of the p53-MDM2 interaction for targeted molecular therapy of malignant neoplasms. Proc Natl Acad Sci U S A. 107: 14321–14326. 10.1073/pnas.1008930107 PMC2922601 20660730

[25] Villalonga-Planells R, Coll-Mulet L, Martínez-Soler F, Castaño E, Acebes JJ, et al (2011) Activation of p53 by nutlin-3a induces apoptosis and cellular senescence in human glioblastoma multiforme. PLoS One 6: e18588. 21483692 10.1371/journal.pone.0018588 PMC3071734

[26] Gomez-Monterrey I, Bertamino A, Porta A, Carotenuto A, Musella S, et al (2010) Identification of the Spiro(oxindole-3,3′-thiazolidine)-Based Derivatives as Potential p53 Activity Modulators. J Med Chem 53: 8319–8329. 21058726 10.1021/jm100838z

[27] Boyum A (1968) Isolation of mononuclear cells and granulocytes from human blood. Isolation of monuclear cells by one centrifugation and of granulocytes by combining centrifugation and sedimentation at 1 g. Scand J Clin Lab Invest Suppl 97: 77–89. 4179068

[28] Lee SK, Kim YC, Song SB, Kim YS (2010) Stabilization and translocation of p53 to mitochondria is linked to Bax translocation to mitochondria in simvastatin-induced apoptosis. Biochem Biophys Res Commun. 391: 1592–1597. 10.1016/j.bbrc.2009.12.077 20043868

[29] Sosin AM, Burger AM, Siddiqi A, Abrams J, Mohammad RM, et al (2012) HDM2 antagonist MI-219 (spiro-oxindole), but not Nutlin-3 (cis-imidazoline), regulates p53 through enhanced HDM2 autoubiquitination and degradation in human malignant B-cell lymphomas. J Hematol Oncol. 5: 57. 10.1186/1756-8722-5-57 PMC3473265 22989009

[30] Vaseva AV, Yallowitz AR, Marchenko ND, Xu S, Moll UM (2011) Blockade of Hsp90 by 17AAG antagonizes MDMX and synergizes with Nutlin to induce p53-mediated apoptosis in solid tumors. Cell Death Dis. 2: e156. 10.1038/cddis.2011.39 PMC3122118 21562588

[31] Vandesompele J, De Paepe A, Speleman F (2002) Elimination of primer-dimer artifacts and genomic coamplification using a two-step SYBR green I real-time RT-PCR. Anal Biochem 303: 95–98. 11906156 10.1006/abio.2001.5564

[32] Chelli B, Lena A, Vanacore R, Da Pozzo E, Costa B, et al (2004) Peripheral benzodiazepine receptor ligands: mitochondrial transmembrane potential depolarization and apoptosis induction in rat C6 glioma cells. Biochem Pharmacol 68: 125–134. 15183124 10.1016/j.bcp.2004.03.008

[33] Ruan S, Okcu MF, Pong RC, Andreeff M, Levin V, et al (1999) Attenuation of WAF1/Cip1 expression by an antisense adenovirus expression vector sensitizes glioblastoma cells to apoptosis induced by chemotherapeutic agents 1,3-bis(2-chloroethyl)-1-nitrosourea and cisplatin. Clin Cancer Res 5: 197–202. 9918219

[34] Chelli B, Rossi L, Da Pozzo E, Costa B, Spinetti F, et al (2005) PIGA (N,N-Di-n-butyl-5-chloro-2-(4-chlorophenyl)indol-3-ylglyoxylamide), a new mitochondrial benzodiazepine-receptor ligand, induces apoptosis in C6 glioma cells. Chembiochem 6: 1082–1088. 15883977 10.1002/cbic.200400350

[35] Dimri GP, Lee X, Basile G, Acosta M, Scott G, et al (1995) A biomarker that identifies senescent human cells in culture and in aging skin in vivo. Proc Natl Acad Sci U S A 92: 9363–9367. 7568133 10.1073/pnas.92.20.9363 PMC40985

[36] Bradford MM (1976) A rapid and sensitive method for the quantitation of microgram quantities of protein utilizing the principle of protein-dye binding. Anal Biochem 7: 248–254. 10.1016/0003-2697(76)90527-3 942051

[37] Sorriento D, Campanile A, Santulli G, Leggiero E, Pastore L, et al (2009) A new synthetic protein, TAT-RH, inhibits tumor growth through the regulation of NFkappaB activity. Mol Cancer 8: 97. 19900276 10.1186/1476-4598-8-97 PMC2780391

[38] Santulli G, Cipolletta E, Sorriento D, Del Giudice C, Anastasio A, et al (2012) CaMK4 Gene Deletion Induces Hypertension. J Am Heart Assoc 1: e001081. 23130158 10.1161/JAHA.112.001081 PMC3487344

[39] Berenbaum MC (1989) What is synergy? Pharmacol Rev. 41: 93–141. 2692037

[40] Tallarida RJ (1992) Statistical analysis of drug combinations for synergism. Pain. 49: 93–97. Review. Erratum in: Pain 1993 53: 365. 10.1016/0304-3959(92)90193-F 1594286

[41] Tallarida RJ, Stone DJ Jr, McCary JD, Raffa RB (1999) Response surface analysis of synergism between morphine and clonidine. J Pharmacol Exp Ther. 289: 8–13. Erratum in: J Pharmacol Exp Ther 1999 289: 1184. 10086981

[42] Tallarida RJ (2002) The interaction index: a measure of drug synergism. Pain. 98: 163–168. 10.1016/s0304-3959(02)00041-6 12098628

[43] Wang CC, Liao YP, Mischel PS, Iwamoto KS, Cacalano NA, et al (2006) HDJ-2 as a target for radiosensitization of glioblastoma multiforme cells by the farnesyltransferase inhibitor R115777 and the role of the p53/p21 pathway. Cancer Res 66: 6756–6762. 16818651 10.1158/0008-5472.CAN-06-0185

[44] Zhao P, Wang D, Gao Y, Yang Z, Li X (1998) Overexpression of MDM2, p53, and NCAM proteins in human radiation-induced skin ulcers. J Environ Pathol Toxicol Oncol 17: 125–127. 9546748

[45] Kondo S, Barnett GH, Hara H, Morimura T, Takeuchi J (1995) MDM2 protein confers the resistance of a human glioblastoma cell line to cisplatin-induced apoptosis. Oncogene 10: 2001–2006. 7761100

[46] Mayo LD, Dixon JE, Durden DL, Tonks NK, Donner DB (2002) PTEN protects p53 from Mdm2 and sensitizes cancer cells to chemotherapy. J Biol Chem 277: 5484–5489. 11729185 10.1074/jbc.M108302200

[47] Tabernero J, Dirix L, Schöffski P, Cervantes A, Lopez-Martin JA, et al (2011) A phase I first-in-human pharmacokinetic and pharmacodynamic study of serdemetan in patients with advanced solid tumors. Clin Cancer Res 17: 6313–6321. 21831953 10.1158/1078-0432.CCR-11-1101

[48] Secchiero P, Barbarotto E, Tiribelli M, Zerbinati C, di Iasio MG, et al (2006) Functional integrity of the p53-mediated apoptotic pathway induced by the nongenotoxic agent nutlin-3 in B-cell chronic lymphocytic leukemia (B-CLL). Blood 107: 4122–4129. 16439677 10.1182/blood-2005-11-4465

[49] Stühmer T, Chatterjee M, Hildebrandt M, Herrmann P, Gollasch H, et al (2005) Nongenotoxic activation of the p53 pathway as a therapeutic strategy for multiple myeloma. Blood 106: 3609–3617. 16081689 10.1182/blood-2005-04-1489

[50] Kojima K, Konopleva M, Samudio IJ, Shikami M, Cabreira-Hansen M, et al (2005) MDM2 antagonists induce p53-dependent apoptosis in AML: implications for leukemia therapy. Blood 106: 3150–3159. 16014563 10.1182/blood-2005-02-0553 PMC1895324

[51] Tovar C, Rosinski J, Filipovic Z, Higgins B, Kolinsky K, et al (2006) Small-molecule MDM2 antagonists reveal aberrant p53 signaling in cancer: implications for therapy. Proc Natl Acad Sci U S A 103: 1888–1893. 16443686 10.1073/pnas.0507493103 PMC1413632

[52] Shangary S, Ding K, Qiu S, Nikolovska-Coleska Z, Bauer JA, et al (2008) Reactivation of p53 by a specific MDM2 antagonist (MI-43) leads to p21-mediated cell cycle arrest and selective cell death in colon cancer. Mol Cancer Ther 7: 1533–1542. 18566224 10.1158/1535-7163.MCT-08-0140 PMC2494594

